# Immunohaemostasis: a new view on haemostasis during sepsis

**DOI:** 10.1186/s13613-017-0339-5

**Published:** 2017-12-02

**Authors:** Xavier Delabranche, Julie Helms, Ferhat Meziani

**Affiliations:** 1 Université de Strasbourg, Faculté de Médecine & Hôpitaux Universitaires de Strasbourg, Service de Réanimation, Nouvel Hôpital Civil, Strasbourg, France; 2 INSERM (French National Institute of Health and Medical Research), UMR 1260, Regenerative Nanomedicine (RNM), FMTS, Université de Strasbourg, Strasbourg, France; 3 INSERM, EFS Grand Est, BPPS UMR-S 949, Université de Strasbourg, Strasbourg, France

**Keywords:** Infection, Septic shock, Disseminated intravascular coagulation (DIC), Host defence peptides (HDPs), Contact phase, Neutrophil extracellular traps (NETs)

## Abstract

Host infection by a micro-organism triggers systemic inflammation, innate immunity and complement pathways, but also haemostasis activation. The role of thrombin and fibrin generation in host defence is now recognised, and thrombin has become a partner for survival, while it was seen only as one of the “principal suspects” of multiple organ failure and death during septic shock. This review is first focused on pathophysiology. The role of contact activation system, polyphosphates and neutrophil extracellular traps has emerged, offering new potential therapeutic targets. Interestingly, newly recognised host defence peptides (HDPs), derived from thrombin and other “coagulation” factors, are potent inhibitors of bacterial growth. Inhibition of thrombin generation could promote bacterial growth, while HDPs could become novel therapeutic agents against pathogens when resistance to conventional therapies grows. In a second part, we focused on sepsis-induced coagulopathy diagnostic challenge and stratification from “adaptive” haemostasis to “noxious” disseminated intravascular coagulation (DIC) either thrombotic or haemorrhagic. Besides usual coagulation tests, we discussed cellular haemostasis assessment including neutrophil, platelet and endothelial cell activation. Then, we examined therapeutic opportunities to prevent or to reduce “excess” thrombin generation, while preserving “adaptive” haemostasis. The fail of international randomised trials involving anticoagulants during septic shock may modify the hypothesis considering the end of haemostasis as a target to improve survival. On the one hand, patients at low risk of mortality may not be treated to preserve “immunothrombosis” as a defence when, on the other hand, patients at high risk with patent excess thrombin and fibrin generation could benefit from available (antithrombin, soluble thrombomodulin) or ongoing (FXI and FXII inhibitors) therapies. We propose to better assess coagulation response during infection by an improved knowledge of pathophysiology and systematic testing including determination of DIC scores. This is one of the clues to allocate the right treatment for the right patient at the right moment.

**Electronic supplementary material:**

The online version of this article (10.1186/s13613-017-0339-5) contains supplementary material, which is available to authorized users.

## Background

The aim of this review is to describe the battle between a foreign pathogen and the host regarding thrombin generation, one of the key molecules to win or to lose the war for surviving. Thrombin is involved in thrombus formation (via fibrin network), in anticoagulation and fibrinolysis [via thrombomodulin and (activated) protein C], focalisation (via glycosaminoglycans and antithrombin), but also in vascular permeability and tone (via endothelial cell receptors and kinin pathways) [1–3].

During infection, initiation of thrombin generation may occur through different pathways [35]:

i. Bacteria initiation with endothelial invasion [4] and platelet activation (via FcγRIIa, αIIbβ3 and platelet factor 4) [5],
ii. Bacterial polyphosphate (polyP) initiation through the “contact” pathway [6],
iii. Endothelial cell expression of encrypted tissue factor (TF), vascular cell recruitment and activation by thrombin, cytokines and microparticles [1, 7, 8],
iv. fibrin network, neutrophil extracellular traps (NETs) and histones [9, 10].

Haemostasis should therefore be considered as a non-specific first line of host defence—at least when localised to a unique endothelial injury—considering the growing role of platelets as immune cells [11–13]. This immune response has been called “immunothrombosis” [14]. In this line, immunohaemostasis process may help to capture pathogens, prevent tissue invasion and concentrate antimicrobial cells and peptides including thrombin-derived host defence peptides. Therefore, when regulated, a low-grade activation of thrombin generation may help survive the bacterial challenge [14]. Yet, inhibition of thrombin generation by Dabigatran promotes bacterial growth and spreading with increased mortality in experimental model of *Klebsiella pneumoniae*-induced murine pneumonia [15].

On the other hand, thrombin can become deleterious if ongoing activation of the coagulation, owing to defective natural anticoagulants, leads to excessive thrombin formation. Combined with defective fibrinolysis, thrombin results in fibrin deposits in microvessels and eventually in disseminated intravascular coagulation (DIC) [16, 17]. DIC thus represents a deregulation and/or an overwhelmed haemostasis activation response triggered by pathogens and/or host responses during septic shock [14]. DIC could be classified in “asymptomatic”, “bleeding” (haemorrhagic), “thrombotic” (organ failure) and ultimately “massive bleeding” (fibrinolytic) type, according to its clinical presentation [18]. Except asymptomatic one, all types are characterised by delayed clotting times (PT and aPTT), low fibrinogen and platelets count owing to their consumption [19, 20]. Although known for many years, the role of DIC in the pathogenesis of septic shock remains a matter of debate [21–23]. Since then, coagulation was considered as a potential therapeutic target. The recognition of new targets implied in thrombosis—but not in haemostasis—opens a new window over innovative therapies.

## Physiology of thrombin generation

For didactic settings, haemostasis can be separated into three phases:

i. Initiation,
ii. Propagation and regulation,
iii. Fibrinolysis.

A brief overview of haemostasis is available in Additional file 1 and Additional file 2: Figure S1 provides the different steps of thrombin generation, fibrin formation and regulation [1, 24].

## Pathophysiology of thrombin and fibrin formation during infection

The contact between a prokaryote and a eukaryote can result in symbiosis or infection resulting in host or pathogen survival. To survive infection, the host initiates a complex inflammatory response including innate immunity, complement and coagulation pathways. These two cascades have a unique origin, but many refinements over the past 500 million years improved their specificities [25, 26]. In this view, coagulation is fundamental to survive and the following section will highlight the role of contact activation system (not involved in “normal” haemostasis), the interplay between pathogens, coagulation and fibrinolysis pathways, and the emerging role of antimicrobial host defence peptides generated by proteolysis of “coagulation” proteins [17, 27, 28].

### Initiation: the emerging role of contact activation system (Fig. 1)

#### Physiology or pathophysiology?

An old view of haemostasis distinguished two initiation pathways: tissue factor (“extrinsic” pathway) and contact activation system (CAS) (“intrinsic” pathway). The latter requires a “contact” activator, prekallikrein (PK), high molecular weight kininogen (HK), factor XII (FXII) and FXI [29]. A deficit of one of these proteins results in prolonged aPTT although no haemorrhagic diathesis is evidenced in patients. CAS does not seem to be involved in “normal” haemostasis and may be restricted to pathological conditions resulting in negatively charged surfaces, including sepsis (via NETs and polyP), but also acute respiratory distress syndrome (ARDS) [30] and blood contact with artificial surfaces (intravascular catheters, extracorporeal circuits).

**Fig. 1:**
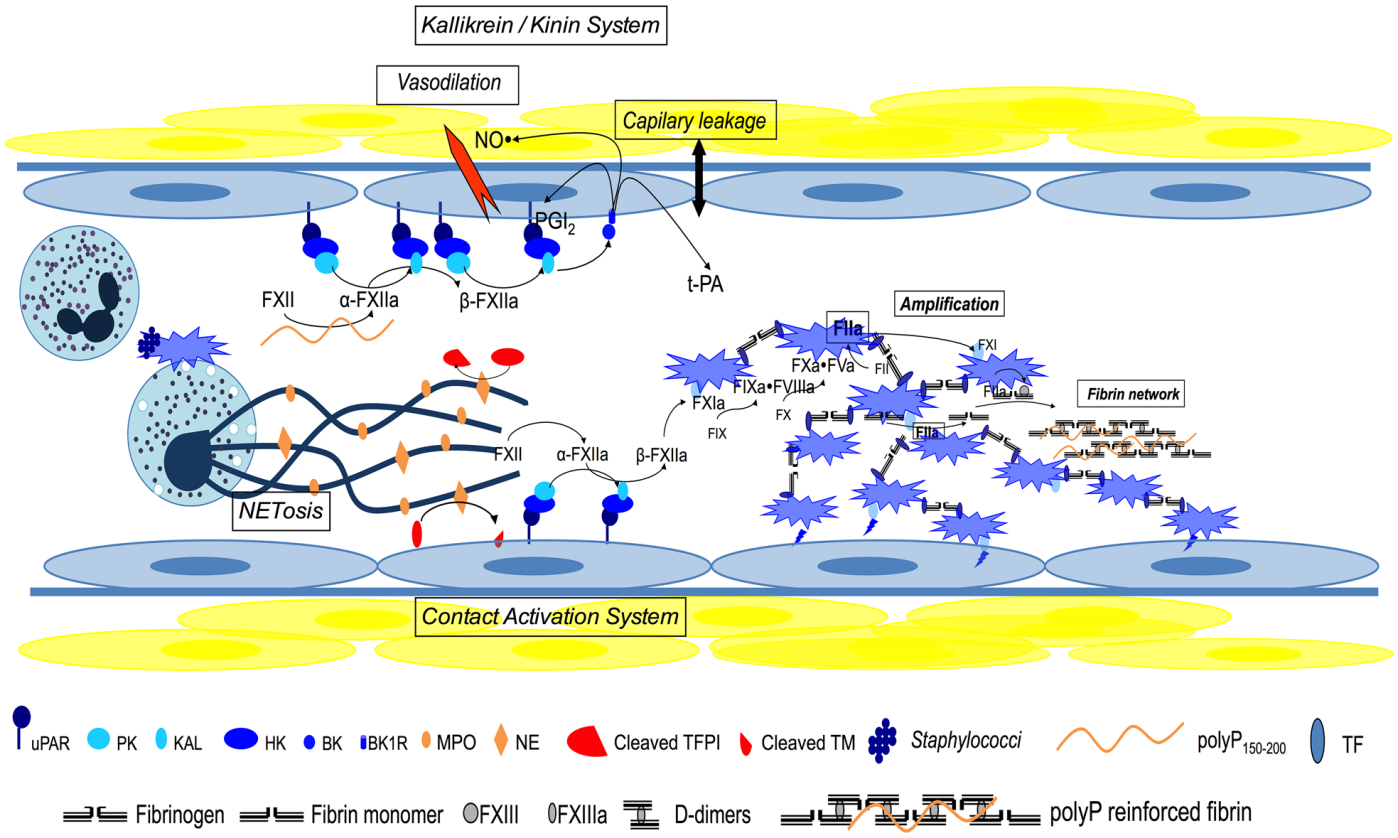
Immunohaemostasis and infection. During infection, bacteria trigger platelet activation via PF4 and TLRs and can initiate neutrophil extracellular traps (NETs) release by neutrophils after chromatin decondensation and nuclear membrane disruption. Negatively charged DNA, decorated with histones, myeloperoxidase (MPO) and neutrophil elastase (NE), is a potent inducer of FXII auto-activation as well as polyphosphates (polyP_150–200_) released by bacteria. Both are “contact” activators, i.e. a negatively charged surface able to link and induce a conformational change in FXII that auto-activates FXII in α-FXIIa in the presence of Zn^2+^. Then α-FXIIa converts PK to kallikrein (KAL) that enables a reciprocal hetero-activation of α-FXII, leading to large amount of β-FXIIa and thereafter platelet GP_Ib_-bound FXI activation. Large amount of FXIIa generated is able to convert platelet-bound FXI into FXIa involved in thrombin generation and fibrin generation. Interestingly, neutrophil elastase (NE) released with NETs is also able to enhance platelet adhesion and activation (inactivation of ADAMTS13) and coagulation with inhibition of tissue factor pathway inhibitor (prolonged tissue factor-induced initiation) and thrombomodulin (impaired activation of protein C). Moreover, polyP_150–200_ enhances activation of platelet-bound FXI by FXIIa and can be incorporated in the fibrin network, reinforcing its structure. On the other hand the kallikrein/kinin system (KKS) is also triggered. FXIIa and KAL convert high molecular weight kininogen (HK) in biologically active bradykinin (BK). BK is not involved in thrombin generation, but mainly in inflammatory response via two G-coupled receptors, B1R and B2R. BK results in increased vascular permeability, vasodilation (mediated by both PGI_2_ and nitric oxide after iNOS induction), oedema formation and ultimately hypotension

“Contact” activator is a negatively charged surface able to link and induce a conformational change in FXII that auto-activates FXII in α-FXIIa in the presence of Zn^2+^. Then α-FXIIa converts PK to kallikrein (KAL) that enable a reciprocal hetero-activation of α-FXII, leading to large amount of β-FXIIa and thereafter platelet GP_Ib_-bound FXI activation. β-FXIIa is also able to activate the classic complement system pathway via C1r and to a lesser extent C1 s linking haemostasis and complement-mediated host defence [3].

CAS and PK also activate fibrinolysis and tissue proteolysis. HK linked to urokinase-type plasminogen activator receptor (uPAR) is able to activate pro-uPA into uPA that in turn activates plasminogen into matrix-bound plasmin. Moreover, BK induces tPA release by endothelial cells when linked to B1R [2].

Besides and related to CAS, the kallikrein/kinin system (KKS) is also activated [3]. CAS and PK also activate fibrinolysis and tissue proteolysis and are regulated by serpin C1 esterase inhibitor (C1-INH). A deficit (responsible for hereditary angioedema) or consumption (during septic shock but also after extracorporeal circulation) is responsible for increased permeability syndrome [31].

#### Polyphosphates (polyP)

PolyP are negatively charged inorganic phosphorous residue polymers, highly conserved in prokaryotes and eukaryotes. They are important source of energy, but are also involved in cell response. Half-life of polyP is very short due to their degradation by phosphatases [32, 33].

Medium-size soluble polyP_60–80_ are released by activated platelets and mast cells. They are able to induce FXII activation only if large amounts are present [34, 35]. PolyP_60–80_ could also bind α-FXIIa preventing further degradation, resulting in prolonged half-life. In the presence of fibrin polymers associated with polyP_60–80_, α-FXIIa can activate fibrin-bound plasminogen in plasmin, resulting in “intrinsic” fibrinolytic activity overcoming antifibrinolytic properties [36, 37]. Interestingly, activated platelets could retain polyP_60–80_ on their surface assembled into insoluble spherical nanoparticles with divalent metal ions (Ca^2+^, Zn^2+^). These nanoparticles provide higher polymer size and become able to trigger contact system activation [38, 39].

On the other hand, large-sized insoluble polyP_150–200_ are released by bacteria and yeasts. PolyP_150–200_ are able to support auto-activation of FXIIa and to promote thrombin generation independently of FXI activation. PolyP can bind FM resulting in clots with reduced stiffness and increased deformability [40]. Moreover, polyP_150–200_ are incorporated in fibrin mesh, inhibiting fibrinolysis [34].

### Neutrophil extracellular traps (NETs)

Neutrophils have long been considered as suicidal cells killing extracellular pathogens. Few years ago, biology of neutrophils has evolved for a more complex network linking innate immunity, adaptive immunity and haemostasis [41–43]. Neutrophils do not only engulf pathogens (phagocytosis) and release granules content, but also release their nuclear content, essentially histones and DNA fragments resulting in a net. These NETs support histones and other granule enzymes like myeloperoxidase (MPO) and neutrophil elastase (NE). These fragments are called NETs for neutrophils extracellular traps, and they enable to trap pathogens and blood cells, including platelets, in their meshes [44].

Two mechanisms of NETosis are described: a suicidal one [44–46] and a vital one, with functional anucleated phagocytic cell survival [47]. Finally, the plasma membrane bursts and NETs are released [48].

NETosis plays a critical role in host defence through innate immunity, but also through other procoagulant mechanisms:

i. Negatively charged DNA constitutes an activated surface for coagulation factors assembly, including contact phase;
ii. Enzymatic inhibition of tissue factor pathway inhibitor (TFPI) and thrombomodulin (TM) by neutrophil elastase;
iii. Direct recruitment and activation of platelets by histones [14].

Recent data support a direct activation by DNA and histones more than NETs themselves [49]. High levels of circulating histones have been evidenced in septic shock. Histone infusion induces intravascular coagulation with thrombocytopenia and increased D-dimers. Antihistone antibodies can prevent both lung and cardiac injuries in experimental models. C-reactive protein can bind histones and reduce histone-induced endothelial cell injury. C-reactive protein infusion rescues histone-challenged mouse [50].

### Pathogen-induced modulation of blood coagulation (Table 1)

#### Initiation of coagulation

All bacteria can induce blood coagulation in a polyP-dependent pathway as seen above. High molecular weight kininogen (HK) can also bind bacterial surface allowing a better activation by host proteases [51]. Interestingly, some bacteria use specific pathways to induce thrombin and fibrin generation [52–58].

**Table 1:** Pathogen-induced modulation of blood coagulation

Bacteria	Protein	Target	Result	References
*A*—*initiation of coagulation*				
All bacteria	PolyP	FXII → FXIIa	Contact phase activation (FXI)	[51]
*S. aureus*	Coagulase	FII → FIIa	Non-proteolytic activation	[52]
	von Willebrand binding protein (vWbp)	vWF (endothelium)	*S. aureus* anchorage to endothelium	[53]
		FII → FIIa	Non-proteolytic activation	[52]
		vWbp-FIIa → FXIII	Clot stabilisation	[53]
	Clumping factor A (ClfA) and fibronectin-binding protein A (FnbpA)	Fg	*S. aureus*—platelet bridging and clot formation	[54]
	Staphopains A and B (ScpA, ScpB)	HK → BK	Vascular leakage	[55, 56]
Group G *streptococci*	Fibrinogen-binding protein (FOG) and protein G (PG)	FXII → FXIIa	Contact phase complex assembly and activation (FXI) at bacterial surface	[57]
*B. anthracis*	Zinc metalloprotease InhA1	FX → FXa/FII → FIIa	Fibrin deposition	[57, 58]
		ADAMTS13 inhibition	Platelet adhesion/activation by UL-vWF	[58]
*B*—*degradation of fibrin clot*				
*B. burgdorferi*	Outer surface proteins (OspA and OspC) and Erp proteins (ErpA, ErpC and ErpP)	Plasmin(ogen)	Plasminogen activation by tPA/uPA	[62]
*H. influenzae*	Surface protein E (PE)	Plasmin(ogen)	Plasminogen activation by tPA/uPA	[63]
*Streptococci spp.*	α-Enolase	Plasmin(ogen)	Plasminogen activation by tPA/uPA	[64–67]
*B. anthracis*	α-Enolase and elongation factor tu	Plasmin(ogen)	Plasminogen activation by tPA/uPA	[66]
*S. pyogenes*	Plasminogen-binding M-like protein (PAM) and streptokinase (SK)	Plasminogen	Direct non-enzymatic activation	[51]
			Metalloprotease activation and tissue invasion by PAM-bound SK·PM	[68, 69]
*S. agalactiae*	Skizzle (SkzL)	tPA/uPA	Enhanced plasminogen activation	[70]
*Y. pestis*	Omptin Pla	Plasminogen	Direct activation in presence of LPS	[71]
		PAI-1/TAFI/α_2_-AP	Inactivation of serpins	[72–74]
*S. enterica*	Omptin PgtE	PAI-1/α_2_-AP	Inactivation of serpins	[76]
*C*—*inactivation of fibrinolysis*				
Group A *streptococci*	Collagen-like proteins (SclA and SclB)	TAFI and FIIa	TAFI → TAFIa	[77, 78]
*D*—*Inhibition of coagulation*				
Group A *streptococci*	Streptococcal inhibitor of complement (SIC)	HK	Inhibition of HK binding and contact phase activation	[79, 80]
*S. aureus*	Staphylococcal superantigen-like protein 10 (SSLP-10)	FII	Inhibition of platelet binding and activation	[81]

#### Degradation of fibrin clots

Fibrin formation and pathogen entrapment are key features of host defence during infection. Fibrin(ogen) is fundamental to survive infection [59]. To evade fibrin, many bacteria developed fibrinolysis activators or expressed plasminogen receptors allowing activation by host tPA or uPA [60–76].

Outer membrane proteins (omptins) are surface-exposed, transmembrane β-barrel proteases exposed by some gram-negative bacteria. They display fibrinolytic and procoagulant activities required for pathogenicity [71, 72]. *Yersinia pestis* is the agent of bubonic and pneumonic plague. Both associate haemorrhagic and thrombotic disorders and the presence of Pla, a direct activator of host plasminogen, require rough LPS. Pla is also able to promote fibrinolysis by activation of uPA, inactivation of serpins PAI-1 and α_2_-antiplasmin and by cleavage of C-terminal region of TAFI with reduced activation by thrombin–thrombomodulin complex [73, 74]. Pla is also able to cleave TFPI. Interestingly, dysplasminogenemia (Ala^601^ → Thr), present in about 2% of the Chinese, Korean and Japanese populations, confers a protection against plague. Homozygous individuals have a reduced plasminogen activity about 10% with fewer thrombotic events, but enhanced survival during infection by *Y. pestis* but also by group A *streptococci* and *S. aureus* requiring plasminogen activation for pathogenicity [75].

#### Inactivation of fibrinolysis

Inhibition of fibrinolysis is another way to promote clot stabilisation [77, 78].

#### Inhibition of coagulation

Bacteria can also block contact activation pathway [79, 80] or thrombin generation [81] in order to prevent host defence.

### Host defence peptides

Innate immunity is mediated by cell activation via Toll-like receptors (TLRs). Resulting cationic and amphipathic small peptides (15–30 amino acids, < 10 kDa) have many biological properties including direct bactericidal effects, but also immunomodulation and angiogenesis. They have been named “host defence peptides” (HDPs) or “antimicrobial peptides” (AMPs).

In eukaryotes, we can identify defensins (disulphide-stabilised peptides) and cathelicidins (α-helical or extended peptides). HDPs can be classified into three categories regarding their target on prokaryotes:

i. Plasma membrane-active peptides disrupting membrane integrity,
ii. Intracellular inhibitors of transcription or translational factors and
iii. Cell wall-active peptides interfering with cell wall synthesis and bacterial replication [82].

Limited proteolysis of many proteins involved in blood coagulation (activators as well as inhibitors) is now recognised as HDPs and may participate to host defence. Interestingly, the development of synthetic HDPs is a new therapeutic anti-infectious strategy regarding resistance of pathogens to (conventional) antibiotics [83].

#### Serine protease-derived peptides

Human serine proteases (including vitamin K-dependent blood coagulation factors and kallikrein system peptides) can be cleaved by proteases to generate C-terminal peptides with direct antimicrobial activities [84]. GKY25 is released from FIIa, FXa and FXIa after cleavage by neutrophil elastase [85]. This peptide is able to slightly reduce *P. aeruginosa* growth but also to significantly reduce both inflammatory response and mortality [86]. Bacteria are also able, mainly by unknown mechanisms, to generate HDPs from fibrinogen (GHR28) and high molecular weight kininogen (HKH20 and NAT26).

#### Serpin-derived peptides

Serpins (or serine protease inhibitors) can also generate HDPs. Heparin cofactor II (HCII) can be cleaved by neutrophil elastase after binding to glycosaminoglycan [87], and KYE28 displays antimicrobial properties against gram-negative and gram-positive bacteria but also against fungus [87]. Moreover, KYE28 can bind LPS dampening inflammatory response [88]. FFF21 derived from antithrombin also shares antimicrobial activity after permeabilisation of bacterial membrane [89]. Protein C inhibitor-derived SEK20 peptide displays antimicrobial activity [90]. Interestingly, platelets can bind PCI under activation resulting in high concentration of PCI at site of platelet recruitment as observed during infection [91].

## Diagnosis

Activation of the coagulation cascade is a physiologic, innate and adaptive response during infection. This response can be overwhelmed, becoming hazardous and referred to as DIC meaning disseminated intravascular coagulation, as well as “death is coming” [92]. For many years, only two conditions were distinguished: “no DIC” and “DIC”. This “schizophrenic” view of haemostasis needs to be reissued, as proposed by Dutt and Toh [93]: “The Ying-Yang of thrombin and protein C”. There is indeed a *continuum* from adaptive to noxious thrombin generation. Moreover, DIC remains a medical paradigm for critical care physicians: clinical diagnosis is often (too) late and biological diagnosis (too) frequent in the absence of clinical signs or therapeutic opportunities [94].

### Clinical diagnosis

Most patients with sepsis and septic shock do not present any clinical sign of “coagulopathy”, while routine laboratory tests are disturbed. Clinical examination should focus on purpura, symmetric ischaemic limb gangrene (with pulses) [95] and diffuse oozing. A very specific sign is “retiform purpura”, which is a netlike purpura reminiscent of livedo. However, unlike classic livedo, in which meshes are erythematous, meshes are here purpuric. The absence of induced bleeding on retrieval when the skin is punctured to a depth of 3 to 4 mm within a livid or purpuric area is a good indication of thrombotic microangiopathy [96].

### Laboratory criteria

A single test will never be able to diagnose and stratify sepsis-induced coagulopathy. Only a combination of the presence of underlying disease associated with evidence of cellular activation in the vascular compartment (including endothelial cells, leucocytes and platelets), procoagulant activation, fibrinolytic activation, inhibitor consumption and end-organ damage or failure will allow such diagnosis.

#### Underlying disease

In sepsis and septic shock, vascular injury is central and prompted by different actors with overlapping kinetics, leading to difficulties in deciphering a sequential order [97].

Acute kidney injury (AKI) is present in about half patients, one-third of non-DIC patients *versus* four-fifth in DIC patients. This association between AKI and low platelets may be symptomatic of thrombotic microangiopathy (TMA) all the more that Ono et al. [98] reported low ADAMTS13 activity and high UL-vWF in septic shock-induced DIC. Nevertheless, there are two important differences: the presence of schizocytes and the absence of prolonged clotting times in TMAs [99, 100].

Hepatic injury is frequent, but remains mild to moderate, with a slight increase in liver enzymes and bilirubin and decrease in PT. On the other hand, severe hepatic ischaemia may lead to fulminant hypoxic hepatitis with very low PT, but also inhibitors AT and PC mimicking DIC with ischaemic limb gangrene with pulses [101].

#### Cellular activation

Only indirect markers of cellular activation are available; most of them are not routinely assessed. These markers could be soluble molecules (released by shedding or by proteolytic cleavage) or cell-derived microvesicles, including microparticles (MPs). The role of MPs in septic shock and infection has been discussed elsewhere [102–104].

##### Endothelial cells

 E-selectin (CD62E), or endothelial-leucocyte adhesion molecule-1 (ELAM-1), is only expressed by endothelial cells after cytokine stimulation. CD62E is involved in leucocyte recruitment at site of injury and could be released in the blood stream as free, soluble molecule (sCD62E) or membrane bound after MP shedding (CD62^+^-MPs). sCD62E is dramatically increased during septic shock, especially in DIC patients [8], but was not associated with DIC diagnosis in one study [105, 106]. Interestingly, CD62^+^-MPs were not increased in septic shock due to proteolysis [8].

Endoglin (CD105, Eng) is a membrane protein expressed mainly by endothelial cells in the vascular repair and angiogenesis during inflammation [107]. It contains an arginine-glycine-aspartic acid (RGD) tripeptide sequence that enables cellular adhesion, through the binding of integrins or other RGD binding receptors that are present in the extracellular matrix. Membrane-bound CD105 is involved in leucocyte α5β1 activation, resulting in leucocyte recruitment and extravasation on the one hand and in angiogenesis on the other hand, whereas MMP-14-cleaved soluble (s)CD105 abolishes extravasation and inhibits angiogenesis [107]. CD105 plays a pivotal role in endothelial cell adhesion to mural cells [108]. Soluble CD105 overexpression is actually linked to other typical systemic and vascular inflammation states, as pre-eclampsia and HELLP syndrome, that are also characterised by a haemostatic activation/deregulation [109] and podocyturia [108]. We evidenced the presence of CD105^+^-MPs during septic shock, especially in DIC patients [8, 110].

Endothelial cells also release soluble and microparticle-bound EPCR. sEPCR is a marker of endothelial injury and severity [111], while EPCR^+^-MPs can display an anticoagulant and cytoprotective pattern in the bloodstream [112, 113].

##### Leucocytes

Neutrophils and monocytes play a major role in sepsis-induced coagulopathy. After stimulation by thrombin and cytokines, monocytes could express TF and promote thrombin generation after cell membrane remodelling and phosphatidylserine (PhtdSer) exposition. Moreover, TF^+^-MPs of monocyte origin have been identified and could disseminate a procoagulant potential [7].

The role of neutrophils is more complex, involving both TF expression (fusion of TF^+^-MPs) [114] and NETs [115]. Direct evidence of the presence of NETs in bloodstream is lacking, but histones (or nucleosomes), free DNA and myeloperoxidase could be detected in plasma and are significantly increased in septic shock-induced DIC [116]. Recently, our group showed cytological modification of neutrophils in blood smears of patients with DIC [117]. Moreover, we evidenced neutrophil chromatin decondensation assessed by measuring neutrophil fluorescence (NEUT-SFL) using a routine automated flow cytometer Sysmex™ XN20 [118].

##### Platelets

Inflammation resulting in systemic inflammatory response syndrome (SIRS) is a potent inducer of both fibrinogen synthesis and platelet circulating pool mobilisation. Platelet count can reach 700–800 G/L, but thrombocytopenia can occur during sepsis. A “normal” value—that is to say in the normal range—may be interpreted cautiously and represent patent consumption. Moreover, enumeration is not function. During sepsis-induced coagulopathy, platelet activation follows thrombin generation and does not support the propagation phase of haemostasis with impaired P-selectin, ADP, Ca^2+^ and cFXIII local supply.

##### Erythrocytes

Schizocytes are fragmented erythrocytes and are the cornerstone of TMA diagnosis. They are frequently observed on blood smears during DIC and remain of poor value for DIC diagnosis [119].

#### Procoagulant activation

Routine coagulation tests evidence a prolongation of both prothrombin time (PT) and activated partial thromboplastin time (aPTT). Nevertheless, PT is the more accurate. aPTT is only slightly elevated during DIC due to inflammatory response and very high level of FVIII released by injured endothelial cells.

Evidence of thrombin generation can be evaluated by quantification of prothrombin fragment 1 + 2 (F1 + 2) and/or thrombin–antithrombin (TAT) complexes. These tests are not routinely available. Moreover, we evidenced the lack of discrimination of F1 + 2 between DIC and non-DIC patients despite significant differences [8].

Fibrin formation is quantified by fibrinopeptide A (FpA) (with a 2:1 ratio), not available in routine [120]. Soluble fibrin monomers (FM) can be routinely quantified. They do not represent fibrin formation, but resting fibrin monomers not yet polymerised by FXIIIa. High FM can evidence increased production and/or defective polymerisation [121, 122]. The accuracy of this biomarker is still matter of debate (see below) [123, 124].

#### Fibrinolytic activation

Fibrin(ogen) degradation products (FDPs) are heterogeneous small molecules generated by the action of plasmin on both fibrin network (secondary fibrinolysis) and fibrinogen (primary fibrinogenolysis). D-dimers (D-domain of two fibrin molecules stabilised by FXIIIa) are specific of fibrinolysis and must be preferred when available [125–127]. D-dimers sign thrombin generation, fibrin formation and polymerisation then fibrinolysis, while the absence of D-dimers could represent defective fibrinolysis despite the presence of fibrin. Other markers could be useful but are not available in routine laboratories: PAP (plasmin–antiplasmin complexes), tPA and PAI-1 [128, 129]. Both tPA and PAI-1 are dramatically increased during septic shock, regardless of DIC diagnosis. Early inhibition of fibrinolysis during sepsis-induced coagulopathy may cause diagnostic delay regarding the importance of FDPs in DIC diagnosis.

#### Inhibitors consumption

Sustained thrombin generation leads to activation, then consumption, of regulatory mechanisms. TFPI is decreased during DIC [130]. Antithrombin can be—and should be—routinely assessed during sepsis-induced coagulopathy. The absence of low AT level challenges the diagnosis of DIC [131]. Concerning the TM-APC pathway, assessment is complex. PC is decreased by consumption, but APC is increased, at least at the beginning of sepsis. Moreover, soluble forms of EPCR (sEPCR) [111] and TM (sTM) [132] can be found in plasma of septic patients and are correlated to vascular injury.

#### Global assessment of haemostasis

Thromboelastography (TEG) and rotational thromboelastometry (ROTEM™) are routinely used in operative theatres to monitor blood coagulation and “assess global haemostasis” [133]. Interestingly, they can also evaluate fibrinolysis at 30 and 60 min. Nevertheless, a recent Cochrane review concluded that there was little or no evidence of the accuracy of such devices, strongly suggesting that they should only be used for research [99, 100]. Few data are available regarding septic shock-induced coagulation/coagulopathy. A prospective study comparing septic shock patients, surgical patients and healthy volunteers evidences a hypocoagulability during DIC [134]. In this study, we may hypothesise that DIC patients were in “fibrinolytic” phase.

#### Scoring systems

Different scoring systems have been developed to ensure DIC diagnosis and are discussed in supplementary data (Additional file 1, Additional file 3: Table S1).

## New therapeutic opportunities?

**Fig. 2:**
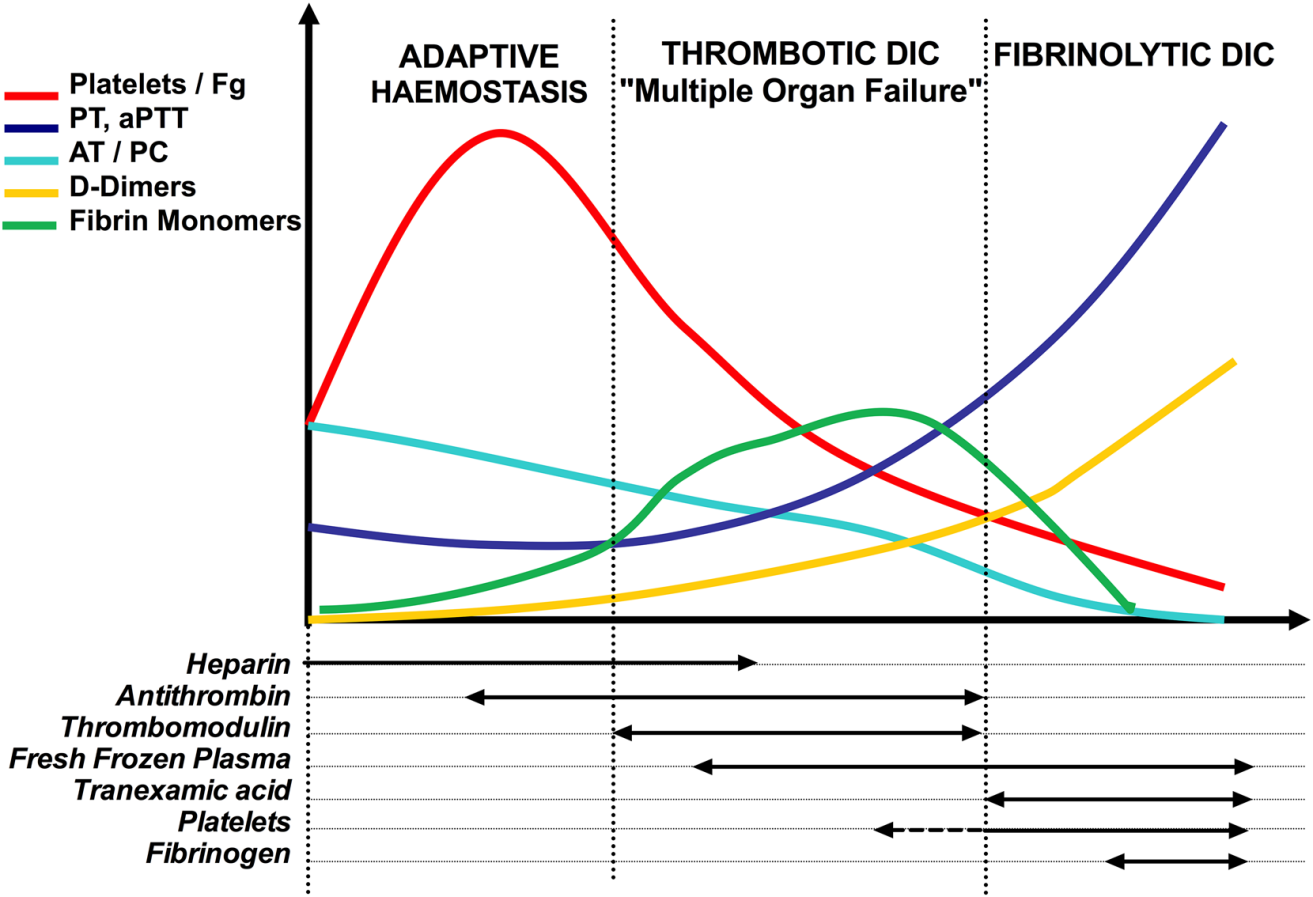
Natural history of coagulation during infection and potential therapeutics. The first step is “adaptive haemostasis” associated with the systemic inflammatory syndrome. Platelet count increases and fibrinogen production is dramatically increased (red curve). Thrombin generation is initiated with slight shortening of PT and aPTT (dark blue curve) resulting in fibrin monomers generation (green curve). Natural anticoagulants, antithrombin and protein C are decreased by consumption and downregulation (light blue curve). Inhibition of fibrinolysis by PAI-1 results in low D-dimers (yellow curve). Only low-dose heparin (unfractionated or low molecular weight) could be recommended to prevent thrombosis (inferior part of the graph). Reduction of anticoagulants and continuous thrombin generation results in prolonged clotting times (PT and aPTT) and platelet and fibrinogen consumption that remain in the high normal range. Fibrin monomers increased due to sustained fibrin formation and defective polymerisation by FXIIIa. D-dimers are moderately increased. This step can be called “thrombotic/multiple organ failure DIC” step and could be treated by natural anticoagulant infusion (antithrombin or soluble thrombomodulin) or fresh-frozen plasma. Later in the natural evolution of coagulation, consumption of all factors and platelets results in very low levels of fibrinogen, AT and PC, prolonged PT and aPTT and massive fibrinolysis with very high D-dimers. This “fibrinolytic DIC” step is characterised by oozing and massive bleeding, and supportive therapy associates fresh-frozen plasma and platelet transfusions, fibrinogen supply and tranexamic acid to prevent fibrinolysis

A syllogism precludes anticoagulant therapy during severe sepsis and septic shock: “more severe is the infection, more thrombin is generated”, “more thrombin is generated, more organ failure and death supervene”, so “more you prevent thrombin generation, more you will improve your patient with severe infection”. This view forgets that haemostasis is mandatory to survive sepsis via many pathways, including newly recognised immunothrombosis and HDPs. In fact, “anticoagulant” treatments disrupt a tight equilibrium between pathogen and adaptive host response and may lead to more deaths in a group of patients (adaptive haemostasis) and to fewer deaths in another group (noxious haemostasis). Recognition of “noxious haemostasis” remains a medical paradigm for critical care physicians. Negative therapeutic interventions [135, 136], drotrecogin alfa withdrawal [137], but also emerging concept of immunothrombosis [14] could argue for a radical “tabula rasa” regarding coagulation during septic shock. The debate is still open and can be summarised in one question: “Should all patients with sepsis receive anticoagulation?” [138, 139]. Finally, whether immunohaemostasis/DIC clinical assessment is reliable remains a major issue (Fig. 2).

A mini-review of current (and past) therapies is provided in supplementary data (Additional file 1, Additional file 4: Table S2, Additional file 5: Table S3 and Additional file 6: Figure S2) regarding:

i. limitation of thrombin and fibrin generation,
ii. DIC with thrombotic/multiple organ failure pattern,
iii. DIC with haemorrhagic pattern.

In the following section, we will present an overview of therapies focused on immunohaemostasis activation.

### Inhibition of contact pathway

Contact pathway is not necessary for “normal” haemostasis. FXII(a) and FXI(a) are new targets to develop “safe” antithrombotic drugs without antihaemostatic effects [140–142]. Moreover, these drugs could improve hypotension targeting bradykinin release.

#### C1-inhibitor

C1-inhibitor regulates both complement activation and FXII and could improve both capillary leakage and hypotension on the one hand and contact phase-induced thrombin generation on the other. As other serpins, C1-inhibitor is dramatically reduced in septic shock and C1-inhibitor supplementation could improve patients or renal function in short randomised trials [143–145]. Nevertheless, no large randomised trial can support its use. Interestingly, bradykinin receptor antagonist icatibant had no effect on a porcine model of septic shock [146].

#### FXII blockade

In a baboon model challenged with a lethal dose of *E. coli*, the monoclonal antibody C6B7 directed against FXIIa improved survival with higher blood pressure. In the treated group, the inflammatory response was reduced with lower IL-6 and neutrophil elastase release as well as complement activation. Inhibition of FXIIa was obvious with reduced BK released and fibrinolysis. Nevertheless, both groups experiment DIC with low platelet count, low fibrinogen and low FV [147]. Another FXIIa monoclonal blocking antibody is 3F7. This antibody seems to be safe as an anticoagulant in experimental extracorporeal membrane oxygenation model, with reduced bleeding compared to heparin, but no data are yet available regarding septic shock [148].

#### FXI blockade

14E11 is an anti-FXI monoclonal antibody that blocks FXI activation by FXIIa but not by FIIa. 14E11 displays antithrombotic properties. This molecule was used in mouse polymicrobial sepsis. Inflammation and coagulopathy were improved as well as survival after 14E11 treatment up to 12 h after bowel perforation onset. Clotting time was not modified, and no bleeding could be evidenced in this model [149].

Interestingly, FXI KO mice (FXI^−/−^) evidence increased inflammatory response with impaired neutrophil functions—but not haemorrhage in lungs—in a model of *Klebsiella pneumoniae* and *Streptococcus pneumoniae* pneumonia resulting in an increased mortality. Inhibition of FXI activation by FXIIa does not reproduce this pattern [150].

A genetically engineered fusion protein (MR1007) containing anti-CD14 antibody (to block LPS receptor) and the modified second domain of bikunin (with anti-FXIa activity) improves survival in a rabbit model of sepsis without increasing spontaneous bleeding [151].

### Inhibition of platelet functions in thrombus formation

Platelets are important immune cells, and thrombocytopenia is associated with an increased mortality in septic shock [152, 153]. Few data support a benefit of previous aspirin treatment in community-onset pneumonia with [154] or without septic shock [155]. In a retrospective study of patients with septic shock, chronic antiplatelet treatment was not associated with reduced mortality [156]. There are no data to support introduction of antiplatelet therapy or to transfuse platelets in the absence of obvious thrombocytopenia with bleeding.

### Inhibition of polyP

Targeting polyP is a new opportunity in the treatment of contact phase-induced thrombosis, including immunothrombosis, but some of them are toxic in vivo and cannot be used in humans (polymyxin B, polyethylenimine and polyamidoamine dendrimers) [157].

#### Universal heparin reversal agents (UHRAs)

UHRAs have been developed to reverse heparin effects but also displayed anticoagulant effects. UHRA-9 and UHRA-10 specifically inhibit polyP and prove antithrombotic effects without increasing bleeding in a mouse model of arterial thrombosis [158]. Nevertheless, these agents have not been used in experimental septic shock to date.

#### Phosphatases

Platelet-derived polyP are rapidly degraded by phosphatases. During septic shock, alkaline phosphatase activity is dramatically decreased and could enhance polyP activity. A recombinant human alkaline phosphatase (RecAP) is able to improve renal function due to acute kidney injury during septic shock [159–161]. Moreover, RecAP inhibits platelet activation ex vivo by converting ADP in adenosine and reverse hyperactivity of septic shock-derived platelets [162]. Effects on polyP were not specifically studied in this experimental study but cannot be excluded.

#### Dabrafenib

Dabrafenib is a B-Raf kinase inhibitor indicated in unresectable or metastatic melanoma with BRAF V600E mutation. This molecule has anti-inflammatory effects on polyP-mediated vascular disruption and cytokine production. In a mouse model of CLP-induced septic shock, administration of Dabrafenib 12 and 50 h after ligation improves survival [163].

### Inhibition of NETs/histones

#### Deoxyribonuclease 1 (DNase 1)

Deoxyribonuclease 1 or dornase alfa (Pulmozyme^®^) is an inhaled potent inhibitor of bacterial DNA used in patients with cystic fibrosis. Few experimental data are available regarding NETs. In a mouse model of thrombosis, DNase 1 infusion disassembles NETs and prevents thrombus formation [164]. Interestingly, in a CLP model of sepsis, DNase 1 delayed—but not early—infusion reduces organ failure and improves outcome [165]. More recently, DNase 1 infusion in mice challenged with LPS, *E. coli* or *S. aureus* reduces thrombin generation and platelet aggregation and improves microvascular perfusion [166] and survival [167].

#### Interferon-λ1/IL-29

IFN-λ1/IL-29 is a potent antiviral cytokine able to prevent NETs release induced by septic shock sera or platelet-derived polyP after phosphorylation of mammalian target of rapamycin (mTOR) to downregulate autophagy. Moreover, IFN-λ1/IL-29 does not alter neutrophil viability and ROS production preserving phagocytosis. IFN-λ1/IL-29 has a strong antithrombotic activity in experimental arterial thrombosis but could also regulate immunohaemostasis [168].

## Conclusion: evidence-based versus pragmatic medicine

Up to date, it is not possible to propose a unique strategy to diagnose and treat coagulation disorders during infection and septic shock. On the one hand, an “old view” considered activation of blood coagulation as one of the principal ways to die and thrombin as the principal suspect. This view was the rationale for anticoagulation during septic shock, with many experimental data supporting it. Nevertheless, all clinical trials—with the exception of PROWESS trial—failed to improve survival in unselected septic shock patients. On the other hand, recent experimental and clinical data support a beneficial role of blood coagulation to survive sepsis, including immunohaemostasis. The first step to improve patients’ care is to stratify the “coagulopathy”. A combination of biological tests must be used daily, eventually combined in scores. We believe that JAAM 2006 and JAAM-DIC scores, taking into account the inflammatory syndrome and evolution, are the most appropriate. New markers of cell activation may be of interest. The second step is the choice of therapeutic intervention. Treatment of both infection and shock without delay is mandatory. Then, anticoagulation may be considered. To date, no recommendation can be made according to international guidelines with a high level of proof. Nevertheless, three different patterns could be recognised (Fig. 2):

i. Absence of obvious coagulopathy with high platelet count, low D-dimers, subnormal PT and AT requiring only prevention of thrombosis by unfractionated or low molecular weight heparins.
ii. Thrombotic/multiple organ failure coagulopathy (also referred as thrombotic DIC) with “low normal” platelet count, prolonged PT, decreased AT and mild to moderate D-dimers level; clinical presentation may combine organ failure and cutaneous signs like symmetric limb gangrene with pulses and retiform purpura. Antithrombin and recombinant soluble thrombomodulin must be considered. New treatments targeting FXIIa, FXIa, polyP and NETs preventing thrombosis are in development and improve survival in experimental sepsis or septic shock. They have not yet been tested in humans.
iii. Haemorrhagic/fibrinolytic coagulopathy with very low platelets, fibrinogen and AT, prolonged coagulation times and clinical oozing. Massive transfusion of fresh-frozen plasma, platelets and fibrinogen is required, with antifibrinolytic drugs.

New clinical trials are necessary to support this view and to improve patients’ care.

## Additional files

**Additional file 1.** Supplementary data.

**Additional file 2: Figure S1.** Physiology of thrombin generation.

**Additional file 3: Table S1.** DIC scoring systems.

**Additional file 4: Table S2.** Efficacy of anticoagulants in septic shock.

**Additional file 5: Table S3.** Effect of antithrombin in pneumonia-induced septic shock with DIC (observational nationwide study)^40^.

**Additional file 6: Figure S2.** Timing of anticoagulant therapy.

## Abbreviations

ADAMTS13: a disintegrin and metalloprotease with thrombospondin type 1 motif
CAS: contact activation system
DIC: disseminated intravascular coagulation
FDPs: fibrin(ogen) degradation products
HDPs: host defence peptides
HK: high molecular weight kallikrein
ISTH: International Society for Thrombosis and Haemostasis
JAAM: Japanese Association for Acute Medicine
KAL: kallikrein
KKS: kallikrein/kinin system
MPO: myeloperoxidase
MPs: microparticles
NE: neutrophil elastase
NETs: neutrophil extracellular traps
PCI: protein C inhibitor
Pg: plasminogen
polyP: polyphosphates
SK: streptokinase
TAFI: thrombin-activatable fibrinolysis inhibitor
TMAs: thrombotic microangiopathies
UL-vWF: ultralarge von Willebrand factor
