# Supplementary material for: Immunohaemostasis: a new view on haemostasis during sepsis
Source: Ann Intensive Care. 2017 Dec 2;7:117. doi: 10.1186/s13613-017-0339-5 (PMC5712298; doi:10.1186/s13613-017-0339-5)
Supplement: Supplementary file 1 — Additional file 1. Supplementary data. [file 13613_2017_339_MOESM1_ESM.docx]

**Immunohaemostasis: a new view on haemostasis during sepsis**

Xavier DELABRANCHE (MD, PhD)^1,2^, Julie HELMS (MD, PhD)^1,3^**,** Ferhat MEZIANI (MD, PhD)^1,2^

^1^Service de Réanimation Médicale, Nouvel Hôpital Civil, Hôpitaux Universitaires de Strasbourg, Strasbourg (France)

^2^EA 7293, Fédération de Médecine Translationnelle de Strasbourg (FMTS), Faculté de médecine, Université de Strasbourg, Strasbourg (France)

^3^INSERM, EFS Grand Est, BPPS UMR-S 949, FMTS, Université de Strasbourg, Strasbourg (France)

**Supplementary data**

**PHYSIOLOGY OF THROMBIN GENERATION**

For a didactic setting, haemostasis can be separate into three phases:

1. initiation,
2. propagation and regulation,
3. fibrinolysis.

Nevertheless, there is an important time – and spatial – heterogeneity in the haemostatic response. Description is out of the scope of this review and could be found elsewhere^1, 2^. Figure 1 briefly represents the different steps of thrombin generation, fibrin formation and regulation.

**Initiation**

In “normal” haemostasis, the two leading events are endothelial denudation with collagen exposure and endothelial cell activation. Collagen is the main activator for platelet recruitment and activation and endothelial cell activation results in the acquisition of a prothrombotic phenotype.

***Platelet activation*** Collagen exposure is responsible of platelet rolling and adhesion (GP_IV_) and activation (GP_VI_). The first step of platelet activation is “outside-in” activation with membrane remodelling including negatively charged phosphatidylserine (PhtdSer) exposure at the membrane outer leaflet. Cytoskeleton proteins are activated resulting in cell contraction and membrane budding, extrusion of granules content and “inside-out” signalling. Platelet-derived microparticles (PMPs) are released during this process and can disseminate a highly procoagulant activity. Moreover, granules content (ADP, prostacyclin or PGI_2_, thromboxane A_2_ or TXA_2_) exerts autocrine amplification effect and paracrine recruitment to neighbouring platelets. GP_IIb-IIIa_ (or integrin α_IIb_β_3_) receptors are activated allowing fibrinogen to link RGD domain. A fibrinogen molecule can link two platelets resulting in platelet aggregation.

***Endothelial cell activation and Tissue Factor expression*** Endothelial cell injury results in glycocalyx rupture and the loss of both anti-adhesive glycosaminoglycans and anticoagulant glycosaminoglycan-bound antithrombin. Membrane remodelling occurs with PhtdSer exposure and endothelial cell-derived MPs (EMPs) emission. Weibel-Palade bodies content (ultralarge von Willebrand factor, UL-vWF and FVIII) are extruded as well as tissue-type plasminogen activator (t-PA) and plasminogen activator inhibitor-1 (PAI-1). Encrypted TF is exposed at the cell membrane in a negatively charged environment allowing (auto)-activation of factor VII (FVII) in active zymogen FVIIa.

***Trace thrombin generation*** Circulating vitamin K-dependent factors bind to PhtdSer *via* Ca^2+^ and concentrate at endothelial cell surface and platelet surface. FVIIa-TF converts few amounts of FX into FXa then FXa converts prothrombin (FII) to thrombin (FIIa). Very few amounts of thrombin are generated close to endothelial injury. FIIa is not able to degrade fibrinogen but is responsible of cofactors activation: FVa (circulating and platelet-derived FV) and FVIIIa (circulating and endothelial cell-derived FVIII). Of note, platelet-derived polyphosphates (containing 60 to 80 phosphate groups, polyP_60-80_) is a potent facilitator of FV activation by thrombin.

Moreover, thrombin can activates neighbouring platelets *via* glycoproteins (GP_Ib-IX-V_) and protease-activated receptors (PAR1 and PAR2). To enhance platelet adhesion, thrombin inhibits ADAMTS13 (A Disintegrin And Metalloprotease with ThromboSpondin type 1 motif) acting as a vWF-cleaving protease. On endothelial cells, PAR1 cleavage by thrombin results in ERK1/2 and RhoA pathways activation and pro-inflammatory response, including vascular leakage.

***Regulation by Tissue Factor Pathway Inhibitor (TFPI)*** Free (unbound) FXa binds protein S (PS) and TFPI, and the trimeric complex TFPI-PS-FXa is able to link TF instead of FVII preventing FVII auto-activation.

**Amplification**

***Josso loop*** GP_Ib_-linked thrombin is able to activate FXI into FXIa in presence of platelet-released polyP_60-80_ then converting FIX into FIXa. Then tenase (FIXa-FVIIIa) converts FX into FXa and FXa with its cofactor FVa results in prothrombinase complex able to convert large amount of prothrombin to thrombin and inactive prothrombin fragment 1+2 (F1+2).

***Fibrin formation and polymerisation*** Fibrinogen-bound FIIa converts one molecule of fibrinogen into (soluble) fibrin monomer (FM), two fibrinopeptides A (FpA) and two fibrinopeptides B. Then FXIIIa is generated after a two-step activation of circulating α_2_β_2_ FXIII – (p)FXIII – by FM-linked thrombin then by low calcium ion while cellular α_2_ isoform (c)FXIII (released by platelets and monocytes) requires only Ca^2+^ to be activated. FXIIIa allows polymerisation of fibrin monomers resulting in a fibrin mesh stabilized by polyP_60-80_.

***Regulation by the thrombomodulin-activated protein C pathway*** Thrombomodulin (TM), expressed by endothelial cell, is another thrombin receptor/cofactor. TM gene expression is up-regulated by heat shock, FIIa, PGE_1_, adenosine and statins, and down-regulated by LPS, TNFα, IL-1β, free fatty acids and endotoxin (LPS). TM can also be degraded by neutrophil elastase, bacteria-derived proteases and cathepsin G.

TM is able to bind FIIa at the EGF-like domain (TMD2) and TM-bound FIIa losses all procoagulant activities and becomes able to cleave protein C (PC) into activated protein C (APC). PC is “presented” by a specific receptor, the Endothelial Protein C Receptor (EPCR). APC can bound its cofactor PS then inactivate FVa (in FVa*_i_*) and FVIIIa (in FVIIIa*_i_*, with FV as a secondary cofactor). Mutation of FV at Arg^506^ (FV^R506Q^ or FV_Leiden_) results in decreased inhibition of FVa by APC and is called “APC resistance”. This heterozygous mutation is associated with mild thrombophilia but also with improved survival during septic shock [^1^](#_ENREF_1). TMD2 is also able to bind uPA promoting its inhibition by FIIa.

Moreover, the N-terminal domain (lectin-like domain, TMD1) inhibits complement activation and displays anti-adhesive properties, limiting neutrophil and monocyte adhesion to endothelium. The serine-threonine rich domain (TMD3, close to the transmembrane domain) enhances FIIa neutralization by protein C inhibitor (PCI), AT and heparin cofactor II (HCII). This domain can bind PF4 that enhances APC and reduces TAFIa generation.

Beside anticoagulant effects, APC has also a potent anti-inflammatory and cytoprotective effects mediated by cell receptors. Briefly, “free” APC can bind endothelial cell ApoE-R2 (increasing Akt), neutrophil β_3_ integrin (inhibiting cell migration) and macrophage CD11b/CD18 (decreasing IL-6 production). Moreover, bound to EPCR, APC is able to cleave PAR1 at a different site than thrombin (Arg^46^ instead of Arg^41^). Non-canonical activation of PAR1, coupled with S1P1, results in vascular protection mediated by β-arrestin, PI3K/Akt and Rac1 pathways. APC is able to modify gene expression with anti-inflammatory and anti-apoptotic properties.

Soluble TM, released after cleavage or recombinant (ART-123, containing TMD1, TMD2 and TMD3 domains), is able to bind FIIa and to promote APC generation resulting in indirect anticoagulant and cytoprotective effects.

***Focalisation by antithrombin*** Free (unbound) thrombin, as well as free FVIIa and FXa, can be captured by antithrombin, a serpin (SERin Protease INhibitor), with thrombin-antithrombin (TAT) inactive complexes. AT activity is greatly enhanced by a cofactor, glycosaminoglycans upon cells or heparins in the blood stream.

AT also exerts anti-inflammatory effects. Some are mediated by anticoagulant properties *via* the prevention of cell activation (platelets and endothelial cells), the suppression of factors promoting neutrophil-endothelial cell interactions (IL-1, IL-6, MCP-1, P-Sel and E-Sel). Others are independent. AT is able to induce PGI_2_ synthesis/secretion by endothelial cells resulting in suppression of platelet activity, inhibition of neutrophil attachment and reduction of released IL-6, IL-8 and TNFα. Moreover, AT is able to bind syndecan-4 receptor on neutrophils, monocytes and lymphocytes, inhibiting their interaction with endothelial cells. Some of these effects are only evidenced with high AT activity, above 200%, not observed in physiology but that could be reached after AT supplementation.

**Fibrinolysis**

***Activation*** Intravascular fibrinolysis requires fibrin-bound plasminogen activation by t-PA. In the blood stream, t-PA is bound to its inhibitor PAI-1 and remains unable to proteolyse plasminogen into plasmin, preventing fibrinogenolysis. t-PA affinity for fibrin is greater than for PAI-1 and the presence of fibrin clot displaces t-PA from PAI-1 to fibrin. Fibrin-bound plasminogen is activated in fibrin-bound plasmin that becomes able to degrade fibrin network into fibrin degradation products, including D-dimers.

***Regulation*** To prevent massive intravascular fibrino(geno)lysis, free plasmin is immediately inhibited by serpins, mainly α_2_-antiplasmin (α_2_-AP or AP) but also C1-convertase inhibitor (C1-INH). In the other hand, fibrinolysis is delayed at site of thrombin formation by the release of large amounts of PAI-1 by injured endothelial cells and by activation of a carboxypeptidase B named TAFI (Thrombin Activable Fibrinolysis Inhibitor) by TM-bound thrombin. TAFIa cleaves fibrin Lys-terminal residues required by plasminogen to link fibrin.

**DISSEMINATED INTRAVASCULAR COAGULATION: SCORING SYSTEMS**

***Historical perspectives*** A “new” condition was characterised during acute promyelocytic leukaemia, obstetrical disorders and other malignant diseases including low platelets, low fibrinogen and prolonged clotting times with both thrombotic and haemorrhagic patterns[^2^](#_ENREF_2)^,^[^3^](#_ENREF_3)^,^[^4^](#_ENREF_4). DIC was born! Nevertheless, DIC was difficult to characterise and no exact diagnostic criteria were available up to the end of the ninety-eighties. Pioneers were Kobayashi in Japan[^5^](#_ENREF_5) and Bick in the United States[^6^](#_ENREF_6), who proposed a combination of clinical signs, underlying disorders and biological tests, resulting in different scores and different classifications. The purpose of these scores was not to predict death, but to evaluate and graduate a (patho)physiologic process and guide therapy. Then, experts from the International Society on Thrombosis and Haemostasis (ISTH) proposed two scoring systems, “overt” DIC and a preceding state called “non-overt” DIC[^7^](#_ENREF_7). The criteria were revised by the Japanese Association for Acute Medicine (JAAM) to take into account inflammatory response during septic shock in 2005[^8^](#_ENREF_8), 2006[^9^](#_ENREF_9) and 2016 following the new definition of septic shock[^10^](#_ENREF_10). Scores are available in Table S1.

***ISTH 2001 “overt” and “non overt”*** These scores were proposed by experts of the Scientific and Standardisation Committee for DIC[^7^](#_ENREF_7). “Overt” DIC was defined by underlying disease AND a score of 5 or more points including platelet count, prothrombin time, FDPs and fibrinogen (Table S1). If “overt” DIC was not present, the patient could be screened for “non overt” DIC. Nevertheless, “non overt” score has never been evaluated during sepsis and septic shock. “Overt” DIC score has been defined regardless underlying disease. Inflammatory syndrome with both thrombocytosis and high fibrinogen, as observed during sepsis, may be a pitfall for DIC diagnosis.

***JAAM 2005, JAAM 2006 and JAAM-DIC 2016*** The Japanese Association for Acute Medicine proposed a new score in 2005 to take into account some pitfalls of ISTH scores during inflammatory disorders characterised by high platelet count and high fibrinogen. The JAAM 2005 score included the presence of SIRS, platelet count, PT, FDPs and fibrinogen (with a “high” cut-off at 3.5 g/L to take into account inflammatory syndrome)[^8^](#_ENREF_8). Nevertheless, fibrinogen was abandoned due to its low predictive value in the 2006 revised JAAM score[^9^](#_ENREF_9). Interestingly, this score is kinetic and must be repeated every day as long as underlying condition remains present. Septic shock criteria and definition was revised in 2015 and SIRS was excluded[^11^](#_ENREF_11). In this view, a new JAAM score has been proposed (JAAM-DIC) replacing SIRS criterion by antithrombin, currently available, with a cut-off value of 70% (Table S1)[^10^](#_ENREF_10).

***Unresolved issues*** There is no “gold standard” for DIC diagnosis and DIC is not unique. It could be a temptation to separate DIC according to underlying disease because pathophysiology, biologic pattern, treatment and prognosis are different (mortality in trauma-induced DIC 12.8% *vs.* 34.7% during sepsis)[^12^](#_ENREF_12). For example, a DIC score in pregnant women has been developed[^13^](#_ENREF_13) and has a better sensitivity than ISTH “overt” score[^14^](#_ENREF_14)^,^[^15^](#_ENREF_15). During septic shock, comparison between ISTH “overt” and JAAM 2006 scores has shown that they were close but not equivalent[^12^](#_ENREF_12). Prospective studies in patients with severe sepsis were published with both scores[^16^](#_ENREF_16)^,^[^17^](#_ENREF_17). These studies evidence a higher mortality when DIC scores increased, ranging from 10-15% (no activation) to 35% (DIC diagnosis) and to more than 45% for highest values[^139^](#_ENREF_139). Substitution of one parameter for another could allow a better assessment of DIC diagnosis. The main pitfall for this approach is the absence of both a “gold standard” for diagnosis and an end-point for prognosis. JAAM was revised with substitution of SIRS score by AT[^10^](#_ENREF_10) and one could propose to use fibrin monomers instead of D-dimers as fibrin-related products[^18^](#_ENREF_18)^,^[^19^](#_ENREF_19)^,^[^20^](#_ENREF_20). Despite increased FM in DIC patients, AUC of ROC curve for FM was only 0.726 (unpublished data from[^8^](#_ENREF_8) ).

***Pre-DIC state assessment*** Some patients will develop DIC during the following hours despite anti-infectious therapies and supportive care. These patients represent about one-third of all-DIC patients and are severely ill with mortality and morbidity as high as DIC patients[^21^](#_ENREF_21)^,^[^22^](#_ENREF_22). Retrospectively, a “pre-DIC” state can be diagnosed the day before the DIC diagnosis was obvious. Early recognition (at admission) could be interesting for specific therapy. This “pre-DIC” state could also be defined as “asymptomatic” DIC[^23^](#_ENREF_23), but is not recognised by ISTH “non overt” DIC criteria. We used microparticles as surrogates of cellular activation in the vascular compartment and evidenced increased endothelial-derived CD105^+^-MPs and leucocyte-derived CD11a^+^-MPs/leucocyte count before DIC was obvious[^21^](#_ENREF_21). A second study confirmed that we were able to predict the absence of asymptomatic DIC using a combination of platelet count, prothrombin time and CD105^+^-MPs[^22^](#_ENREF_22).

**MANAGEMENT**

**Limitation of thrombin and fibrin generation**

This clinical “challenge” has never been specifically studied in clinical trials. The absence of patient’s stratification for “anticoagulant” treatment allocation during severe sepsis and septic shock must be outlined. Nevertheless, indirect response could be helpful. In some *post-hoc* analyses of randomised clinical trials, it is obvious that less seriously injured patients (predicted mortality less than 25%, APACHE II score less than 25 or SAPS 2 less than 40) did not benefit of antithrombin[^24^](#_ENREF_24), activated protein C[^25^](#_ENREF_25) or thrombomodulin[^26^](#_ENREF_26) therapies.

Thrombotic prophylaxis may be sufficient for these patients (heparin and/or intermittent pneumatic compression). Accurate biological monitoring every day could be of interest to diagnose evolution to DIC. Kinetic analyses of platelet count, PT, antithrombin and D-dimers may help clinicians to treat the patient.

**Disseminated intravascular coagulation with thrombotic / multiple organ failure pattern**

In such patients, DIC should be diagnosed according DIC-JAAM 2016 and/or ISTH 2001 “overt” scores. Natural anticoagulants seem to be the best option in the absence of bleeding or urgent surgery[^27^](#_ENREF_27).

***Heparin*** Although commonly used, only six trials have been designed to assess heparin infusion in septic shock – not in DIC (Table S2) [^28^](#_ENREF_28)^,^[^29^](#_ENREF_29). Most trials evidenced a mild to moderate risk of bleeding and heparin cannot be currently recommended in the absence of a large, placebo-controlled trial.

***Antithrombin*** Low AT level (<40%) is frequently evidenced during septic shock and results of down-regulation and consumption[^30^](#_ENREF_30). Interestingly, antithrombin displays both anticoagulant and cytoprotective properties[^31^](#_ENREF_31). Case reports, particularly during meningococcal disease and *purpura fulminans*, support AT supplementation[^32^](#_ENREF_32). Both short prospective clinical and experimental studies were associated with reduced mortality in selected patients, mainly in DIC patients[^33^](#_ENREF_33)^,^[^34^](#_ENREF_34). Antithrombin is currently available and approved in this indication, even if not recommended by the last Sepsis Surviving Campaign guidelines[^35^](#_ENREF_35). Antithrombin monitoring is routinely available and could help to guide therapy and diagnose DIC according to JAAM-DIC score[^10^](#_ENREF_10). Antithrombin supplementation remains essential during heparin anticoagulation of intravascular medical devices or extracorporeal circuits with a target above 60%.

Most randomised prospective trials using antithrombin were not designed to stratify patients according the presence of DIC or not. A recent review for Cochrane library does not support AT substitution in critically ill patients; results are also negative in the subgroup of patients with severe sepsis and DIC (Table S2)[^36^](#_ENREF_36)^,^[^37^](#_ENREF_37).

In Kybersept trial, overall results are negative[^38^](#_ENREF_38). Nevertheless, previously designed *post-hoc* analyses confirm a reduction of 28-day mortality in DIC patients without concomitant infusion of high dose heparin due to increased bleeding complications[^39^](#_ENREF_39), and in intermediate risk of death patients[^24^](#_ENREF_24). Moreover, an observational Japanese nationwide study evidences a benefit of AT in patients with severe pneumonia and DIC (Table S3). AT was associated with a 9.9% [3.1 to 16.3]-reduction in 28-day mortality in this group of patients[^40^](#_ENREF_40).

In trials, AT was used in a large range from 30 to 100 U/kg/day up to 4 days. In a small, non-randomised trial, comparing two doses of AT (1,500 *vs.* 3,000 U/day for 3 days) in patients with septic shock-induced DIC with AT<40%, the higher dose was associated with increased survival without an increased risk of bleeding[^41^](#_ENREF_41).

AT supplementation cannot be recommended in septic shock patients[^35^](#_ENREF_35). Nevertheless, AT is required if extracorporeal circuits are used when AT level is low with concomitant circuit thrombosis despite heparin use[^42^](#_ENREF_42). Moreover, AT could be considered as an “optional” treatment in septic shock-induced DIC with limb gangrene[^43^](#_ENREF_43) and maybe during pneumonia[^40^](#_ENREF_40).

***Activated protein C*** Recombinant human activated protein C (rhAPC) (drotrecogin alfa [activated], Xigris^®^, Eli Lilly Inc., Indianapolis, IN, USA) was indicated between 2002 and 2011 in septic shock patients with two or more organ failures (Europe) or APACHE II score above 25 (USA and Canada), but was not approved in Japan[^44^](#_ENREF_44)^,^[^45^](#_ENREF_45). Impairment of haemostasis was neither an inclusion criteria nor an end-point. rhAPC was withdrawn after negative results of the second randomised trial PROWESS-SHOCK[^45^](#_ENREF_45). Despite similar drug and design, these two trials are not comparable due to highly significant clinical and statistical heterogeneity[^46^](#_ENREF_46). Interestingly, a meta-analysis including randomised and open trials with more than 40,000 patients favours rhAPC (Table S2)[^47^](#_ENREF_47). Moreover, post-hoc analysis of PROWESS patients evidenced benefits in DIC patients in term of coagulation improvement and of 28-day mortality (71/233 *versus* 95/221, *p*=0.006, RR 0.71 [0.55-0.91]; n=454)[^48^](#_ENREF_48). Heterozygous FV_Leiden_ carriers displayed improved survival in PROWESS trial regardless of treatment allocation[^1^](#_ENREF_1). Similar results have been reported in murine sepsis[^49^](#_ENREF_49). Interestingly, trials in one organ-failure patients (ADDRESS)[^25^](#_ENREF_25) or with prolonged infusion up to 168 hours (EXTEND) if shock was not resolved after 96 hours failed to improve survival[^50^](#_ENREF_50).

rhAPC has an anticoagulant activity with prolonged aPTT ratio but also a cytoprotective effect. Experimental data support a major contribution of cytoprotective effects in survival rather than anticoagulation[^51-53^](#_ENREF_51). Interestingly, recombinant APC variants have been genetically produced. The lack of anticoagulant effect allows higher doses enhancing cytoprotection. These modified APCs have been tried in experimental strokes, but not yet in septic shock[^54^](#_ENREF_54)

***Recombinant soluble thrombomodulin*** TM is a transmembrane protein able to bind FIIa with both anticoagulant and anti-inflammatory effects[^55^](#_ENREF_55)^,^[^56^](#_ENREF_56). A soluble recombinant form (ART-123, Recomodulin^®^, Asahi Kasei Pharma Corp., Tokyo, Japan) has been approved in Japan for both sepsis-induced and tumour-induced DIC in 2008[^57^](#_ENREF_57). The therapeutic schedule is 60 µg/kg for 30 min. (up to 6 mg) every day for 6 days.

Japanese studies evidenced an improved 28-day survival in treated patients witha higher rate of DIC resolution at day 7[^58^](#_ENREF_58). In a meta-analysis including 571 patients, mortality was significantly improved (Table S2)[^59^](#_ENREF_59). Nevertheless, analysis in phase II trials was not significant (Table S2). Increased bleeding was not evidenced (*p*=0.33)[^60^](#_ENREF_60). An international randomised, placebo-controlled phase III trial is on-going (NCT#01598831) in septic shock-induced coagulopathy defined by platelet count between 30 and 150 G/L and INR>1.4.

***TFPI*** Recombinant TFPI (Tifacogin) is a potent inhibitor of initiation of haemostasis *via* inhibition of auto-activation of FVII by TF. Phase III clinical trials in septic shock failed to improve survival (Table S2). This drug was withdrawn[^61^](#_ENREF_61).

***Fresh Frozen Plasma (FFP)*** The use of FFP for fluid resuscitation during septic shock has not been evaluated and is not recommended[^35^](#_ENREF_35). Nevertheless, FFP is an important source of procoagulant factors as well as inhibitors and could be of interest before surgery or invasive procedure when clotting times are prolonged. A small prospective study including 60 patients reports the use of FFP in 34 (57%) septic shock patients mainly to prevent bleeding during medical procedures. DIC diagnosis was unknown and mortality not different between the groups[^62^](#_ENREF_62). Incidence of FFP transfusion was 13.7% in few studies[^8^](#_ENREF_8)^,^[^109^](#_ENREF_109), with higher use in DIC patients (37/133 *vs.* 11/218, *p*<0.01).

***Platelet transfusion*** Platelet transfusion is only recommended before surgery, invasive therapy or haemorrhagic disorders, as it might support thrombus formation. The emerging role platelets in innate immunity and experimental data could support platelet transfusion as immune cells[^63-66^](#_ENREF_63). Platelet transfusion improves survival in platelet-depleted animals with pneumonia and sepsis[^67^](#_ENREF_67).

**Disseminated intravascular coagulation with haemorrhagic pattern (fibrinolysis form)**

Spontaneous haemorrhages (oozing) are rare and late during the septic shock course. They are characterised by extreme consumption of all factors and platelets with extremely low platelet count (<20 G/L), very low fibrinogen (<1 g/L), prolonged clotting times (PT and aPTT) and evidence of high fibrinolysis (D-dimers>20 mg/L and euglobulin lysis time (von Kaulla) < 15 min.). JAAM-DIC 2016 and ISTH 2001 “overt” scores are very elevated.

Treatment is supportive requiring massive transfusion of FFP and platelets concentrates, but also fibrinogen. Antifibrinolytic therapy (tranexamic acid) could be of interest. Results are however disappointing and death remains very frequent[^68-70^](#_ENREF_68).

**REFERENCES**

**1.** Kerlin BA, Yan SB, Isermann BH, et al. Survival advantage associated with heterozygous factor V Leiden mutation in patients with severe sepsis and in mouse endotoxemia. *Blood* 2003; **102**(9): 3085-92.

**2.** Rodriguez-Erdmann F. Bleeding due to increased intravascular blood coagulation. Hemorrhagic syndromes caused by consumption of blood-clotting factors (consumption-coagulopathies). *N Engl J Med* 1965; **273**(25): 1370-8.

**3.** Merskey C, Johnson AJ, Kleiner GJ, Wohl H. The defibrination syndrome: clinical features and laboratory diagnosis. *Br J Haematol* 1967; **13**(4): 528-49.

**4.** Lasch HG, Heene DL, Huth K, Sandritter W. Pathophysiology, clinical manifestations and therapy of consumption-coagulopathy ("Verbrauchskoagulopathie"). *Am J Cardiol* 1967; **20**(3): 381-91.

**5.** Kobayashi N, Maekawa T, Takada M, Tanaka H, Gonmori H. Criteria for diagnosis of DIC based on the analysis of clinical and laboratory findings in 345 DIC patients collected by the Research Committee on DIC in Japan. *Bibl Haematol* 1983; **49**: 265-75.

**6.** Bick RL, Ismail Y, Baker WF, Jr. Coagulation abnormalities in patients with precocious coronary artery thrombosis and patients failing coronary artery bypass grafting and percutaneous transcoronary angioplasty. *Semin Thromb Hemost* 1993; **19**(4): 412-7.

**7.** Taylor FB, Jr., Toh CH, Hoots WK, et al. Towards definition, clinical and laboratory criteria, and a scoring system for disseminated intravascular coagulation. *Thromb Haemost* 2001; **86**(5): 1327-30.

**8.** Gando S, Wada H, Asakura H, et al. Evaluation of new Japanese diagnostic criteria for disseminated intravascular coagulation in critically ill patients. *Clin Appl Thromb Hemost* 2005; **11**(1): 71-6.

**9.** Gando S, Iba T, Eguchi Y, et al. A multicenter, prospective validation of disseminated intravascular coagulation diagnostic criteria for critically ill patients: comparing current criteria. *Crit Care Med* 2006; **34**(3): 625-31.

**10.** Iba T, Di Nisio M, Thachil J, et al. Revision of the Japanese Association for Acute Medicine (JAAM) disseminated intravascular coagulation (DIC) diagnostic criteria using antithrombin activity. *Crit Care* 2016; **20**: 287.

**11.** Singer M, Deutschman CS, Seymour CW, et al. The Third International Consensus Definitions for Sepsis and Septic Shock (Sepsis-3). *JAMA* 2016; **315**(8): 801-10.

**12.** Gando S, Saitoh D, Ogura H, et al. Natural history of disseminated intravascular coagulation diagnosed based on the newly established diagnostic criteria for critically ill patients: results of a multicenter, prospective survey. *Crit Care Med* 2008; **36**(1): 145-50.

**13.** Erez O, Novack L, Beer-Weisel R, et al. DIC score in pregnant women--a population based modification of the International Society on Thrombosis and Hemostasis score. *PLoS One* 2014; **9**(4): e93240.

**14.** Jonard M, Ducloy-Bouthors AS, Fourrier F. Comparison of Two Diagnostic Scores of Disseminated Intravascular Coagulation in Pregnant Women Admitted to the ICU. *PLoS One* 2016; **11**(11): e0166471.

**15.** Erez O. Disseminated intravascular coagulation in pregnancy - Clinical phenotypes and diagnostic scores. *Thromb Res* 2017; **151 Suppl 1**: S56-S60.

**16.** Bakhtiari K, Meijers JC, de Jonge E, Levi M. Prospective validation of the International Society of Thrombosis and Haemostasis scoring system for disseminated intravascular coagulation. *Crit Care Med* 2004; **32**(12): 2416-21.

**17.** Gando S, Saitoh D, Ogura H, et al. A multicenter, prospective validation study of the Japanese Association for Acute Medicine disseminated intravascular coagulation scoring system in patients with severe sepsis. *Crit Care* 2013; **17**(3): R111.

**18.** Gris JC, Faillie JL, Cochery-Nouvellon E, Lissalde-Lavigne G, Lefrant JY. ISTH overt disseminated intravascular coagulation score in patients with septic shock: automated immunoturbidimetric soluble fibrin assay vs. D-dimer assay. *J Thromb Haemost* 2011; **9**(6): 1252-5.

**19.** Gris JC, Bouvier S, Cochery-Nouvellon E, Faillie JL, Lissalde-Lavigne G, Lefrant JY. Fibrin-related markers in patients with septic shock: individual comparison of D-dimers and fibrin monomers impacts on prognosis. *Thromb Haemost* 2011; **106**(6): 1228-30.

**20.** Dempfle CE, Wurst M, Smolinski M, et al. Use of soluble fibrin antigen instead of D-dimer as fibrin-related marker may enhance the prognostic power of the ISTH overt DIC score. *Thromb Haemost* 2004; **91**(4): 812-8.

**21.** Delabranche X, Boisrame-Helms J, Asfar P, et al. Microparticles are new biomarkers of septic shock-induced disseminated intravascular coagulopathy. *Intensive Care Med* 2013; **39**(10): 1695-703.

**22.** Delabranche X, Quenot JP, Lavigne T, et al. Early Detection of Disseminated Intravascular Coagulation During Septic Shock: A Multicentre Prospective Study. *Crit Care Med* 2016; **17**: 17.

**23.** Wada H, Matsumoto T, Yamashita Y. Diagnosis and treatment of disseminated intravascular coagulation (DIC) according to four DIC guidelines. *J Intensive Care* 2014; **2**(1): 2052-0492.

**24.** Wiedermann CJ, Hoffmann JN, Juers M, et al. High-dose antithrombin III in the treatment of severe sepsis in patients with a high risk of death: efficacy and safety. *Crit Care Med* 2006; **34**(2): 285-92.

**25.** Abraham E, Laterre PF, Garg R, et al. Drotrecogin alfa (activated) for adults with severe sepsis and a low risk of death. *N Engl J Med* 2005; **353**(13): 1332-41.

**26.** Yoshimura J, Yamakawa K, Ogura H, et al. Benefit profile of recombinant human soluble thrombomodulin in sepsis-induced disseminated intravascular coagulation: a multicenter propensity score analysis. *Crit Care* 2015; **19**(78): 015-0810.

**27.** Fourrier F, Jourdain M, Tournois A, Caron C, Goudemand J, Chopin C. Coagulation inhibitor substitution during sepsis. *Intensive Care Med* 1995; **21**(2): S264-8.

**28.** Zeerleder S, Caliezi C, van Mierlo G, et al. Administration of C1 inhibitor reduces neutrophil activation in patients with sepsis. *Clin Diagn Lab Immunol* 2003; **10**(4): 529-35.

**29.** Zarychanski R, Abou-Setta AM, Kanji S, et al. The efficacy and safety of heparin in patients with sepsis: a systematic review and metaanalysis. *Crit Care Med* 2015; **43**(3): 511-8.

**30.** Fourrier F, Chopin C, Goudemand J, et al. Septic shock, multiple organ failure, and disseminated intravascular coagulation. Compared patterns of antithrombin III, protein C, and protein S deficiencies. *Chest* 1992; **101**(3): 816-23.

**31.** Levy JH, Sniecinski RM, Welsby IJ, Levi M. Antithrombin: anti-inflammatory properties and clinical applications. *Thromb Haemost* 2016; **115**(4): 712-28.

**32.** Fourrier F, Lestavel P, Chopin C, et al. Meningococcemia and purpura fulminans in adults: acute deficiencies of proteins C and S and early treatment with antithrombin III concentrates. *Intensive Care Med* 1990; **16**(2): 121-4.

**33.** Fourrier F, Chopin C, Huart JJ, Runge I, Caron C, Goudemand J. Double-blind, placebo-controlled trial of antithrombin III concentrates in septic shock with disseminated intravascular coagulation. *Chest* 1993; **104**(3): 882-8.

**34.** Inthorn D, Hoffmann JN, Hartl WH, Muhlbayer D, Jochum M. Antithrombin III supplementation in severe sepsis: beneficial effects on organ dysfunction. *Shock* 1997; **8**(5): 328-34.

**35.** Rhodes A, Evans LE, Alhazzani W, et al. Surviving Sepsis Campaign: International Guidelines for Management of Sepsis and Septic Shock: 2016. *Intensive Care Med* 2017; **43**(3): 304-77.

**36.** Allingstrup M, Wetterslev J, Ravn FB, Moller AM, Afshari A. Antithrombin III for critically ill patients. *Cochrane Database Syst Rev* 2016; **8**(2).

**37.** Allingstrup M, Wetterslev J, Ravn FB, Moller AM, Afshari A. Antithrombin III for critically ill patients: a systematic review with meta-analysis and trial sequential analysis. *Intensive Care Med* 2016; **42**(4): 505-20.

**38.** Warren BL, Eid A, Singer P, et al. Caring for the critically ill patient. High-dose antithrombin III in severe sepsis: a randomized controlled trial. *JAMA* 2001; **286**(15): 1869-78.

**39.** Kienast J, Juers M, Wiedermann CJ, et al. Treatment effects of high-dose antithrombin without concomitant heparin in patients with severe sepsis with or without disseminated intravascular coagulation. *J Thromb Haemost* 2006; **4**(1): 90-7.

**40.** Tagami T, Matsui H, Horiguchi H, Fushimi K, Yasunaga H. Antithrombin and mortality in severe pneumonia patients with sepsis-associated disseminated intravascular coagulation: an observational nationwide study. *J Thromb Haemost* 2014; **12**(9): 1470-9.

**41.** Iba T, Saitoh D, Wada H, Asakura H. Efficacy and bleeding risk of antithrombin supplementation in septic disseminated intravascular coagulation: a secondary survey. *Crit Care* 2014; **18**(5): 014-0497.

**42.** Sniecinski RM, Levy JH. Anticoagulation management associated with extracorporeal circulation. *Best Pract Res Clin Anaesthesiol* 2015; **29**(2): 189-202.

**43.** Warkentin TE, Pai M. Shock, acute disseminated intravascular coagulation, and microvascular thrombosis: is 'shock liver' the unrecognized provocateur of ischemic limb necrosis?: *J Thromb Haemost* 2016; **14**(2): 231-5.

**44.** Bernard GR, Vincent JL, Laterre PF, et al. Efficacy and safety of recombinant human activated protein C for severe sepsis. *N Engl J Med* 2001; **344**(10): 699-709.

**45.** Ranieri VM, Thompson BT, Barie PS, et al. Drotrecogin alfa (activated) in adults with septic shock. *N Engl J Med* 2012; **366**(22): 2055-64.

**46.** Kalil AC, Florescu DF. Severe sepsis: are PROWESS and PROWESS-SHOCK trials comparable? A clinical and statistical heterogeneity analysis. *Crit Care* 2013; **17**(4): 167.

**47.** Kalil AC, LaRosa SP. Effectiveness and safety of drotrecogin alfa (activated) for severe sepsis: a meta-analysis and metaregression. *Lancet Infect Dis* 2012; **12**(9): 678-86.

**48.** Dhainaut JF, Yan SB, Joyce DE, et al. Treatment effects of drotrecogin alfa (activated) in patients with severe sepsis with or without overt disseminated intravascular coagulation. *J Thromb Haemost* 2004; **2**(11): 1924-33.

**49.** Kerschen E, Hernandez I, Zogg M, Maas M, Weiler H. Survival advantage of heterozygous factor V Leiden carriers in murine sepsis. *J Thromb Haemost* 2015; **13**(6): 1073-80.

**50.** Dhainaut JF, Antonelli M, Wright P, et al. Extended drotrecogin alfa (activated) treatment in patients with prolonged septic shock. *Intensive Care Med* 2009; **35**(7): 1187-95.

**51.** Mosnier LO, Zlokovic BV, Griffin JH. The cytoprotective protein C pathway. *Blood* 2007; **109**(8): 3161-72.

**52.** Xu J, Ji Y, Zhang X, Drake M, Esmon CT. Endogenous activated protein C signaling is critical to protection of mice from lipopolysaccaride-induced septic shock. *J Thromb Haemost* 2009; **7**(5): 851-6.

**53.** Griffin JH, Zlokovic BV, Mosnier LO. Protein C anticoagulant and cytoprotective pathways. *Int J Hematol* 2012; **95**(4): 333-45.

**54.** Mosnier LO, Zlokovic BV, Griffin JH. Cytoprotective-selective activated protein C therapy for ischaemic stroke. *Thromb Haemost* 2014; **112**(5): 883-92.

**55.** Ito T, Maruyama I. Thrombomodulin: protectorate God of the vasculature in thrombosis and inflammation. *J Thromb Haemost* 2011; **9 Suppl 1**: 168-73.

**56.** Okamoto T, Tanigami H, Suzuki K, Shimaoka M. Thrombomodulin: a bifunctional modulator of inflammation and coagulation in sepsis. *Crit Care Res Pract* 2012; **614545**(10): 28.

**57.** Saito H, Maruyama I, Shimazaki S, et al. Efficacy and safety of recombinant human soluble thrombomodulin (ART-123) in disseminated intravascular coagulation: results of a phase III, randomized, double-blind clinical trial. *J Thromb Haemost* 2007; **5**(1): 31-41.

**58.** Aikawa N, Shimazaki S, Yamamoto Y, et al. Thrombomodulin alfa in the treatment of infectious patients complicated by disseminated intravascular coagulation: subanalysis from the phase 3 trial. *Shock* 2011; **35**(4): 349-54.

**59.** Eguchi Y, Gando S, Ishikura H, et al. Post-marketing surveillance data of thrombomodulin alfa: sub-analysis in patients with sepsis-induced disseminated intravascular coagulation. *J Intensive Care* 2014; **2**(1): 2052-0492.

**60.** Vincent JL, Ramesh MK, Ernest D, et al. A randomized, double-blind, placebo-controlled, Phase 2b study to evaluate the safety and efficacy of recombinant human soluble thrombomodulin, ART-123, in patients with sepsis and suspected disseminated intravascular coagulation. *Crit Care Med* 2013; **41**(9): 2069-79.

**61.** Abraham E, Reinhart K, Opal S, et al. Efficacy and safety of tifacogin (recombinant tissue factor pathway inhibitor) in severe sepsis: a randomized controlled trial. *JAMA* 2003; **290**(2): 238-47.

**62.** Reiter N, Wesche N, Perner A. The majority of patients in septic shock are transfused with fresh-frozen plasma. *Dan Med J* 2013; **60**(4).

**63.** de Stoppelaar SF, van 't Veer C, van der Poll T. The role of platelets in sepsis. *Thromb Haemost* 2014; **112**(4): 666-77.

**64.** Smyth SS, McEver RP, Weyrich AS, et al. Platelet functions beyond hemostasis. *J Thromb Haemost* 2009; **7**(11): 1759-66.

**65.** Semple JW, Freedman J. Platelets and innate immunity. *Cell Mol Life Sci* 2010; **67**(4): 499-511.

**66.** Duerschmied D, Bode C, Ahrens I. Immune functions of platelets. *Thromb Haemost* 2014; **112**(4): 678-91.

**67.** van den Boogaard FE, Schouten M, de Stoppelaar SF, et al. Thrombocytopenia impairs host defense during murine Streptococcus pneumoniae pneumonia. *Crit Care Med* 2015; **43**(3): 0000000000000853.

**68.** Levi M, Toh CH, Thachil J, Watson HG. Guidelines for the diagnosis and management of disseminated intravascular coagulation. British Committee for Standards in Haematology. *Br J Haematol* 2009; **145**(1): 24-33.

**69.** Wada H, Matsumoto T, Yamashita Y. Diagnosis and treatment of disseminated intravascular coagulation (DIC) according to four DIC guidelines. *J Intensive Care* 2014; **2**(1): 15.

**70.** Asakura H. Classifying types of disseminated intravascular coagulation: clinical and animal models. *J Intensive Care* 2014; **2**(1): 2052-0492.

**Figure S1.** Physiology of thrombin generation

1. Initiation: endothelial lesion
2. Initiation: platelet adhesion and activation, tissue factor expression and FVII activation
3. Initiation: trace thrombin generation and FV/FVIII activation
4. Initiation: regulation by tissue factor pathway inhibitor (TFPI)
5. Amplification: platelet FXI activation by trace thrombin and massive thrombin generation (Josso’s loop)
6. Amplification: fibrin formation and fibrin network
7. Amplification: inhibition of thrombin generation by thrombomodulin / activated protein C pathway (TM/APC)
8. Amplification: focalisation by antithrombin
9. Fibrinolysis

**Figure S2.** Timing of anticoagulant therapy

Initially, thrombin generation and SIRS are required for survival, and no therapeutic intervention is mandatory. Specific anticoagulant like activated protein C (Drotrecogin alfa [activated], Xigris^®^) could become deleterious by inhibiting normal, physiologic response. Later, excessive thrombin generation become obvious with decreased natural anticoagulation and pharmacologic supply could be of interest. But when massive consumption of all coagulation factors are evidenced with massive fibrinolysis, an anticoagulant will increase the risk of bleeding and become deleterious.
