# Supplementary figures and images for: Immunohaemostasis: a new view on haemostasis during sepsis
Source: Ann Intensive Care. 2017 Dec 2;7:117. doi: 10.1186/s13613-017-0339-5 (PMC5712298; doi:10.1186/s13613-017-0339-5)

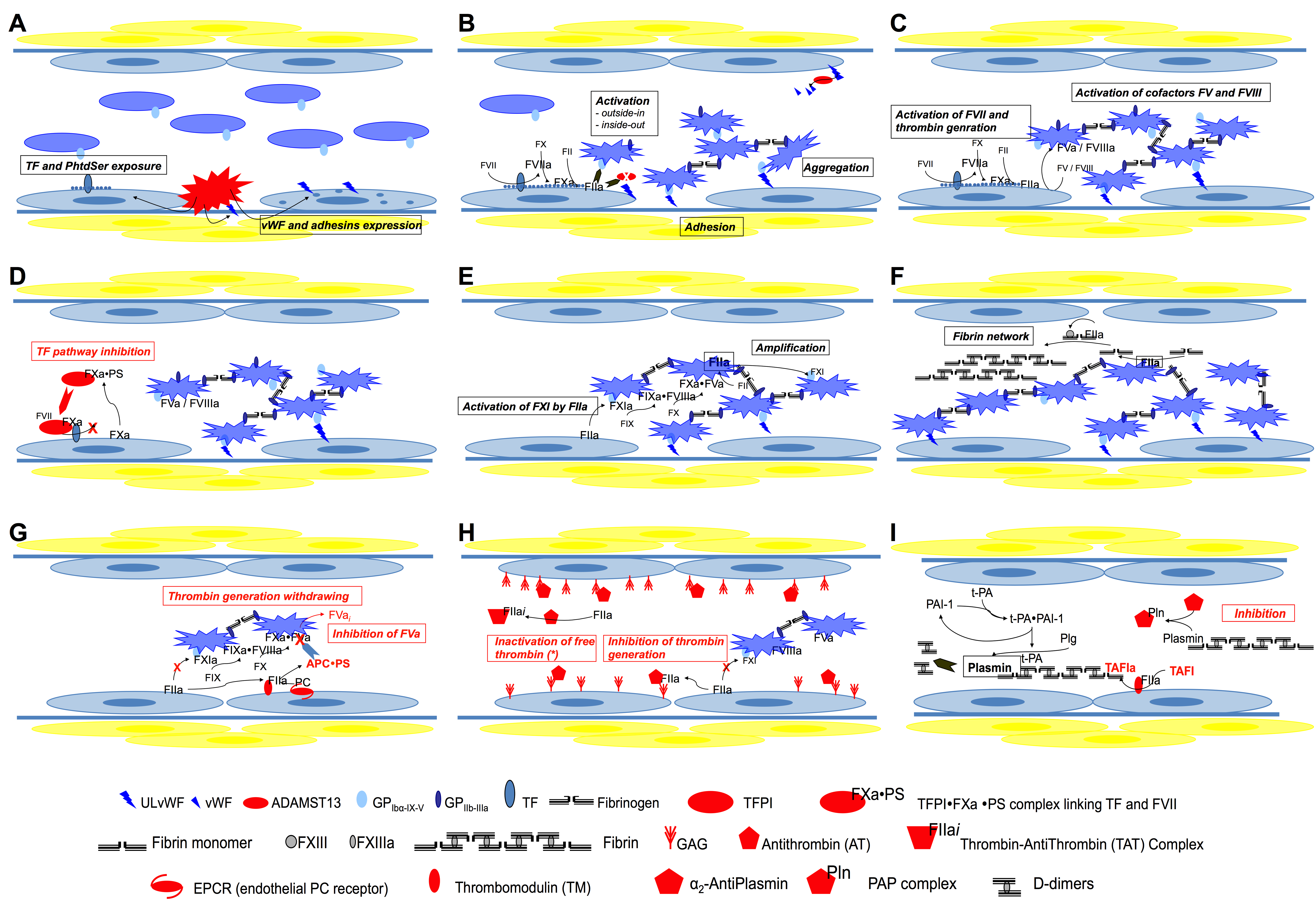

Supplement: Supplementary file 2 — Additional file 2: Figure S1. Physiology of thrombin generation. [file 13613_2017_339_MOESM2_ESM.tiff]

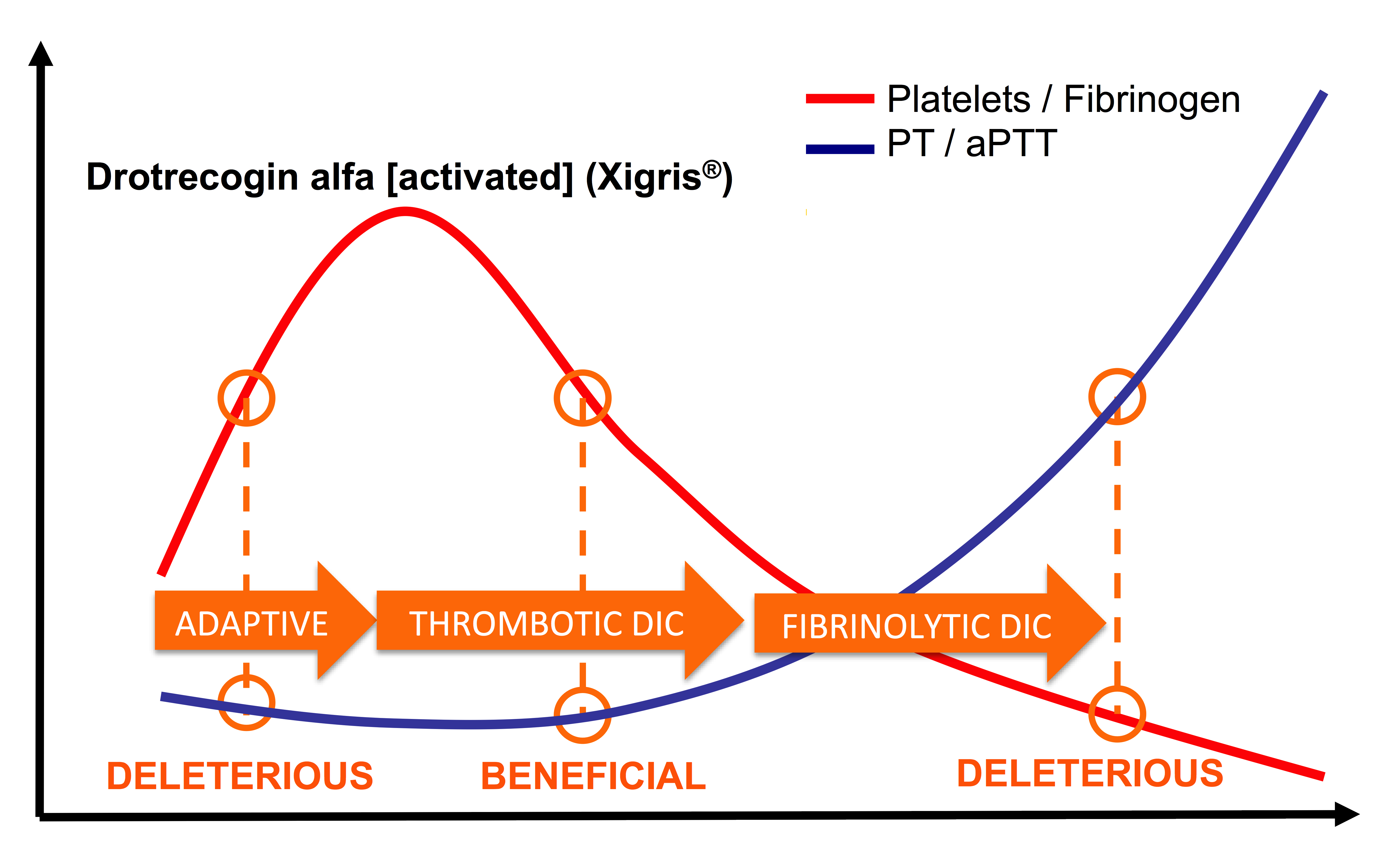

Supplement: Supplementary file 6 — Additional file 6: Figure S2. Timing of anticoagulant therapy. [file 13613_2017_339_MOESM6_ESM.tiff]
