# Supplementary material for: Immunohaemostasis: a new view on haemostasis during sepsis
Source: Ann Intensive Care. 2017 Dec 2;7:117. doi: 10.1186/s13613-017-0339-5 (PMC5712298; doi:10.1186/s13613-017-0339-5)
Supplement: Supplementary file 3 — Additional file 3: Table S1. DIC scoring systems. [file 13613_2017_339_MOESM3_ESM.docx]

**Table S1.** DIC scoring systems

***A – JMWH 1987***^5^

| **Underlying disease** | | | |  |
| --- | --- | --- | --- | --- |
| Absent | 0 point |  |  |  |
| Present | 1 point |  |  |  |
| **Clinical manifestations** | | | |  |
| Bleeding | 1 point | 0 point if haematopoietic malignancies | |  |
| Organ failure | 1 point |  |  |  |
| **Platelet count (G/L)** | | 0 point if haematopoietic malignancies | |  |
| ≥120 | 0 point |  |  |  |
| 80-119 | 1 point |  |  |  |
| 50-79 | 2 points |  |  |  |
| <50 | 3 points |  |  |  |
| **Prothrombin Time ratio** (patient/control) | | **Prothrombin Time (% activity)** | |  |
| <1.25 | 0 point | >60 | 0 point |  |
| 1.24-1.67 | 1 point | 35-60 | 1 point |  |
| ≥1.67 | 2 points | <35 | 2 points |  |
| **Fibrinogen (g/L)** | |  | |  |
| >1.5 | 0 point |  |  |  |
| 1.0-1.4 | 1 point |  |  |  |
| <1.0 | 2 points |  |  |  |

| **Fibrin Degradation Products (mg/L)** | |  | |
| --- | --- | --- | --- |
| <10 | 0 point |  |  |
| 10-19 | 1 point |  |  |
| 20-39 | 2 points |  |  |
| ≥40 | 3 points |  |  |
| ***DIC if 7 points or more OR 4 points or more if haematopoietic malignancies*** | | | |

***B – ISTH 2001 “Overt”***^7^

| **Platelet count (G/L)** | |  |  |
| --- | --- | --- | --- |
| ≥100 | 0 point |  |  |
| 50-99 | 1 point |  |  |
| <50 | 2 points |  |  |
| **Prothrombin Time prolongation (sec)** | | **Prothrombin Time (% activity)** | |
| <3 | 0 point | >64 | 0 point |
| 3-6 | 1 point | 40-63 | 1 point |
| >6 | 2 points | <40 | 2 points |
| **Fibrinogen (g/L)** | |  | |
| >1.0 | 0 point |  |  |
| ≤1.0 | 1 point |  |  |
| **Fibrin Markers** | | **D-dimers (mg/L)** | |
| No change | 0 point | <0.4 | 0 point |
| Moderate | 2 points | 0.4-4.0 | 2 points |
| Strong | 3 points | >4.0 | 3 points |
| ***Overt DIC if 5 points or more*** | | | |

***C – ISTH 2001 “Non Overt”***^7^

| **Underlying disease associated with DIC** (including severe sepsis and septic shock) | | | |
| --- | --- | --- | --- |
| No | 0 point |  |  |
| Yes | +2 points |  |  |
| **Platelet count (G/L)** | |  | |
| ≥100 | 0 point |  |  |
| <100 | +1 point |  |  |
| **Prothrombin Time prolongation (sec)** | | **Prothrombin Time (% activity)** | |
| <3 | 0 point | >64 | 0 point |
| ≥3 | +1 point | ≤64 | +1 point |
| **Fibrin Markers** | | **D-dimers (mg/L)** | |
| No change | –1 point | ≤4.0 | –1 point |
| Increased | +1 point | >4.0 | +1 point |
| **Antithrombin** | | **Antithrombin (% activity)** | |
| Normal | –1 point | ≥70 | –1 point |
| Decreased | +1 point | <70 | +1 point |
| **Kinetic approach** (all parameters, daily) | |  | |
| Improved | –1 point |  |  |
| Stable | 0 point |  |  |
| Worsen | +1 point |  |  |
| ***Non-Overt DIC if 5 points or more. Repeat daily and confirm with ‘Overt’ score.*** | | | |

***D – JAAM-DIC 2016***^10^

| **Antithrombin (%)** | | | |
| --- | --- | --- | --- |
| ≥70 | 0 point |  |  |
| <70 | 1 point |  |  |
| **Platelet count (G/L)** | | **Platelet count, daily reduction** | |
| ≥120 | 0 point |  |  |
| 80-119 | 1 point | **–**30% | 1 point |
| <80 | 3 points | **–**50% | 3 points |
| **Prothrombin Time ratio** (patient/control) | | **Prothrombin Time (% activity)** | |
| <1.2 | 0 point | >64 | 0 point |
| ≥1.2 | 1 point | ≤64 | 1 point |
| **Fibrin Degradation Products (mg/L)** | | **D-dimers (mg/L)** | |
| <10 | 0 point | <5.0 | 0 point |
| 10-25 | 1 point | 5.0-15.0 | 1 point |
| ≥25 | 3 points | ≥15.0 | 3 points |
| ***Overt DIC if 4 points or more. Repeat daily.*** | | | |

***E – R.L. Bick 1993***^6^

|  | **High values** | | | | | **Normal** | **Low Values** | | | |
| --- | --- | --- | --- | --- | --- | --- | --- | --- | --- | --- |
|  | **+4 points** | **+3 points** | **+2 points** | **+1 point** | | **0 point** | **+1 point** | **+2 points** | **+3 points** | **+4 points** |
| **Fibrinopeptide A (µg/L)** | >70 | 41-70 | 11-40 | 3-10 | | <3 | – | – | – | – |
| **Prothrombin F1+2 (nM)** | >10 | 7.5-10 | 6.0-7.4 | 2.7-5.9 | | 0.2-2.6 | – | – | – | – |
| **D-dimers (mg/L)** | >3.0 | 2.0-3.0 | 1.0-2.0 | 0.5-1.0 | | <0.5 | – | – | – | – |
| **FDPs (mg/L)** | >120 | 81-120 | 41-80 | 10-40 | | <10 | – | – | – | – |
| **Antithrombin (%)** | – | – | – | – | | 85-125 | 75-85 | 65-74 | 54-64 | <54 |
| **α_2_-antiplasmin (%)** | – | – | – | – | | 75-120 | 65-74 | 55-64 | 45-54 | <45 |
| **Fibrinogen (g/L)** | – | – | – | – | | 1.50-4.00 | 1.00-1.49 | 0.75-0.99 | 0.50-0.74 | <0.50 |
| **Platelet count (G/L)** | – | – | – | – | | 150-400 | 100-149 | 75-99 | 50-74 | <50 |
| **Temperature (°C)** | >41.0 | 39.0-40.9 | – | 38.5-38.9 | | 36.0-38.4 | 34.0-35.9 | 32.0-33.9 | 30.0-31.9 | <30.0 |
| **MAP (mmHg)** | >160 | 130-159 | 110-129 | – | | 70-109 | – | 50-69 | – | <50 |
| **Heart Rate (b/min.)** | >180 | 140-179 | 110-139 | – | | 70-109 | – | 55-69 | 40-54 | <40 |
| **Repiratory Rate (b/min.)** | >50 | 35-49 | – | 25-34 | | 12-24 | 10-11 | 6-9 | – | <6 |
|  | **High values** | | | | |  | **Low Values** | | | |
|  | **+4 points** | **+3 points** | **+2 points** | **+1 point** | | **0 point** | **+1 point** | **+2 points** | **+3 points** | **+4 points** |
| **PaO_2_ (mmHg)** | – | – | – | – | | 80-100 | 70-79 | 60-69 | 55-60 | <55 |
| **pH** | >7.70 | 7.60-7.70 | – | 7.50-7.59 | | 7.33-7.49 | – | 7.25-7.32 | 7.15-7.24 | <7.15 |
| **Creatinine (µM)** | >308 | 176-307 | 132-175 | – | | 53-131 | – | <53 | – | – |
| **LDH (U/L)** | >275 | 251-275 | 226-250 | 194-255 | | <193 | – | – | – | – |
| **Albumin (g/L)** | – | – | – | – | | 35-55 | 30-34 | 25-29 | 20-24 | <20 |
| **Sodium (mM)** | >180 | 160-179 | 155-159 | 150-154 | | 130-149 | – | 120-129 | 110-119 | <110 |
| **Potassium (mM)** | >7.0 | 6.0-6.9 | – | 5.5-5.9 | | 3.5-5.4 | 3.0-3.4 | 2.5-2.9 | – | <2.5 |
| **Haematocrit (%)** | >60.0 | – | 50.0-59.9 | 46.0-49.9 | | 30.0-45.9 | – | 20.0-29.9 | – | <20.0 |
| **White Blood Cell (G/L)** | >40.0 | – | 20.0-39.9 | 15.0-19.9 | | 3.0-14.9 | – | 1.0-2.9 | – | <1.0 |
| **Column Total (CT)** |  |  |  |  | |  |  |  |  |  |
| ***DIC score (100 – CT) ≥ 90*** | | | | | ***⇨ DIC is unlikely*** | | | | | |
| ***DIC score (100 – CT) = 75-89*** | | | | | ***⇨ DIC is mild*** | | | | | |
| ***DIC score (100 – CT) = 50-74*** | | | | | ***⇨ DIC is moderate*** | | | | | |
| ***DIC score (100 – CT) < 49*** | | | | | ***⇨ DIC is severe*** | | | | | |
