# Supplementary material for: Immunohaemostasis: a new view on haemostasis during sepsis
Source: Ann Intensive Care. 2017 Dec 2;7:117. doi: 10.1186/s13613-017-0339-5 (PMC5712298; doi:10.1186/s13613-017-0339-5)
Supplement: Supplementary file 4 — Additional file 4: Table S2. Efficacy of anticoagulants in septic shock. [file 13613_2017_339_MOESM4_ESM.docx]

**Table S2.** Efficacy of anticoagulants in septic shock

| **Drug** | **RR** | **95% CI** | **I^2^** | **Patients** | **Reference** |
| --- | --- | --- | --- | --- | --- |
| **Heparin (meta-analysis)** | 0.88 | [0.77 – 1.00] | 0% | 2,477 | ^29^ |
| **AT (meta-analysis)** | 0.95 | [0.88 – 1.03] | 0% | 3,882 | ^36^ |
| **AT (subgroup DIC)** | 0.95 | [0.88 – 1.03] | 0% | 2,858 | ^37^ |
| **rhAPC (meta-analysis)** | 0.82 | [0.78 – 0.87] | 40% | 41,401 | ^47^ |
| **rhsTM (meta-analysis)** | 0.59 | [0.45 – 0.77] | 0% | 571 | ^59^ |
| **rhsTM (prospective)** | 0.81 | [0.62 – 1.06] | 0% | 838 | ^60^ |
| **rhTFPI (meta-analysis)** | 0.99 | [0.89 – 1.09] | 0% | 4,267 | ^61^ |
