# Supplementary material for: Immunohaemostasis: a new view on haemostasis during sepsis
Source: Ann Intensive Care. 2017 Dec 2;7:117. doi: 10.1186/s13613-017-0339-5 (PMC5712298; doi:10.1186/s13613-017-0339-5)
Supplement: Supplementary file 5 — Additional file 5: Table S3. Effect of antithrombin in pneumonia-induced septic shock with DIC (observational nationwide study)40. [file 13613_2017_339_MOESM5_ESM.docx]

**Table S3.** Effect of antithrombin in pneumonia-induced septic shock with DIC (observational nationwide study)^40^

| **Analysis** | **Mortality (%)** | | **Patients** | ***p*** | **OR** | **95% CI** | ***p*** |
| --- | --- | --- | --- | --- | --- | --- | --- |
|  | **AT** | **Placebo** |  |  |  |  |  |
| Unmatched | 40.8 | 45.7 | 9,075 | <0.001 | 0.87 | [0.78 – 0.97] | 0.01 |
| Propensity-matched | 40.6 | 44.2 | 4,388 | 0.02 | 0.85 | [0.75 – 0.97] | 0.02 |
| Inverse probability | 41.1 | 45.1 | 18,112 | <0.001 | 0.85 | [0.79 – 0.90] | <0.001 |
